# Impact of chemotherapy for breast cancer on leukocyte DNA methylation landscape and cognitive function: a prospective study

**DOI:** 10.1186/s13148-019-0641-1

**Published:** 2019-03-12

**Authors:** Song Yao, Qiang Hu, Sarah Kerns, Li Yan, Adedayo A. Onitilo, Jamal Misleh, Kelley Young, Lianlian Lei, Javier Bautista, Mostafa Mohamed, Supriya G. Mohile, Christine B. Ambrosone, Song Liu, Michelle C. Janelsins

**Affiliations:** 1 Department of Cancer Prevention and Control, Roswell Park Comprehensive Cancer Center, Buffalo, NY USA; 2 Department of Biostatistics and Bioinformatics, Roswell Park Comprehensive Cancer Center, Buffalo, NY USA; 3 Department of Radiation Oncology, University of Rochester, Rochester, NY USA; 4 Wisconsin NCORP, Weston, WI USA; 5 Delaware NCORP, Newark, DE USA; 6 Kansas City NCORP, Kansas City, KS USA; 7 Department of Public Health Sciences, University of Rochester, Rochester, NY USA; 8 Department of Medicine, University of Rochester, Rochester, NY USA; 9 Department of Surgery, Cancer Control, University of Rochester, Rochester, NY USA; 10 James P Wilmot Cancer Institute, Rochester, NY USA

**Keywords:** DNA methylation, Chemotherapy, Breast cancer, Cognitive function

## Abstract

**Background:**

Little is known about the effects of chemotherapeutic drugs on DNA methylation status of leukocytes, which may be predictive of treatment benefits and toxicities. Based on a prospective national study, we characterize the changes in leukocyte DNA methylome from pre- to post-chemotherapy (approximately 4 months apart) in 93 patients treated for early stage breast cancer and 48 matched non-cancer controls. We further examined significant methylation changes with perceived cognitive impairment, a clinically significant problem related to cancer and chemotherapy.

**Results:**

Approximately 4.2% of the CpG sites measured using the Illumina 450K methylation array underwent significant changes after chemotherapy (*p* < 1e-7), in comparison to a stable DNA methylome in controls. Post-chemotherapy, the estimated relative proportions of B cells and CD4^+^ T cells were decreased by a median of 100% and 39%, respectively, whereas the proportion of monocytes was increased by a median of 91%. After controlling for leukocyte composition, 568 CpGs from 460 genes were still significantly altered following chemotherapy. With additional adjustment for chemotherapy regimen, cumulative infusions, growth factors, and steroids, changes in four CpGs remained significant, including cg16936953 in *VMP1*/*MIR21*, cg01252023 in *CORO1B*, cg11859398 in *SDK1*, and cg19956914 in *SUMF2*. The most significant CpG, cg16936953, was also associated with cognitive decline in breast cancer patients.

**Conclusions:**

Chemotherapy profoundly alters the composition and DNA methylation landscape of leukocytes in breast cancer patients. Our results shed light on the epigenetic response of circulating immune cell populations to cytotoxic chemotherapeutic drugs and provide possible epigenetic links to the degeneration of cognitive function associated with chemotherapy.

**Electronic supplementary material:**

The online version of this article (10.1186/s13148-019-0641-1) contains supplementary material, which is available to authorized users.

## Introduction

Chemotherapy remains an important treatment modality for patients with breast cancer who are at high risk of recurrence [1]. Cytotoxic drugs are often acutely immunosuppressive, negatively affecting the differentiation and viability of leukocytes. Because epigenetic regulation, mainly DNA methylation, drives hematopoiesis and is highly responsive to internal and external insults, it has been widely speculated that chemotherapy would have a profound impact on DNA methylation of blood leukocytes. This effect could influence treatment efficacy, toxicity outcomes, and symptom trajectories. Liquid biopsies of global systemic effects could provide a non-invasive approach to understanding the impact of chemotherapy on the post-treatment outcomes and could help predict those at risk of adverse events [2]. A recent study of ovarian cancer patients who received platinum-based chemotherapy by Flanagan et al. showed that leukocyte DNA methylation changes at relapse were related to overall patient survival [3].

A detailed characterization of the epigenetic changes on leukocyte DNA methylome induced by chemotherapy has been lacking. The Flanagan study assessed DNA methylation at diagnosis and at the time of recurrence; the time intervals between these events varied across patients [3]. Another study compared DNA methylation between two different patient populations, including those treated with chemotherapy and those untreated, and thus could not assess epigenetic changes within the same patients [4]. The goal of this study was to profile the epigenome-wide alterations in leukocyte DNA methylation in peripheral blood of breast cancer patients before and shortly after the completion of chemotherapy. A second group of women who did not receive chemotherapy and had two blood samples collected at a similar time interval were used as a temporal control. Detailed treatment factors that could distinctly alter methylation patterns were considered. We then related significant methylation changes to cognitive complaints, an important clinical problem for breast cancer patients as we previously showed [5], yet we still have limited knowledge of the biologic underpinnings of this problem.

## Results

**Fig. 1:**
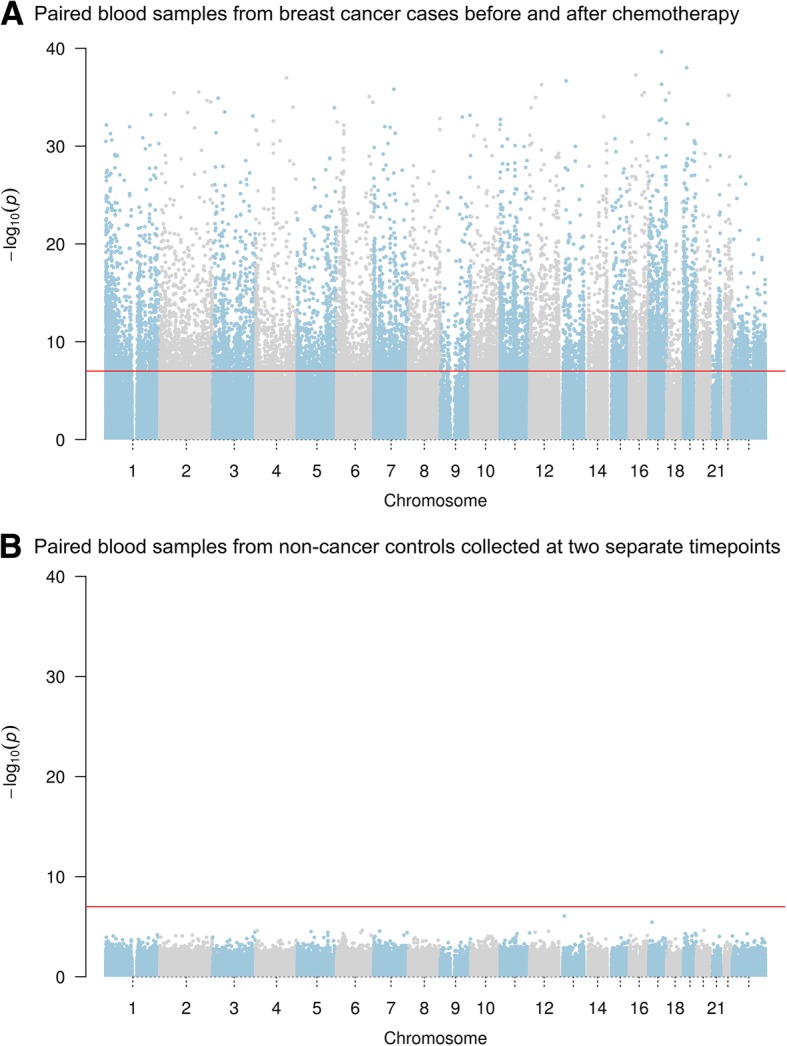
Manhattan plots of changes in leukocyte DNA methylome. Beta-values of each CpG site was compared between paired samples collected from the same individual at two separate time points, and *p* values from paired test were log-transformed and plotted based on chromosomal position. The red line indicates the cutoff significance level of 1e-7 after adjusting for approximately 500,000 tests. **a** Paired peripheral blood samples collected from breast cancer patients at pre- and post-chemotherapy time points with a median of 128 days apart (range 71–230 days). **b** Paired peripheral blood samples collected from healthy controls at two separate time points with a median of 130 days apart (range 42–453 days)

Chemotherapy had a marked impact on leukocyte DNA methylome. Between paired samples collected from breast cancer patients pre- and post-chemotherapy with a median of 128 days apart (IQR 113–157 days), significant changes in methylation value were found at a total of 16,679 CpG sites, or 4.2% of the CpG probes tested, across the methylome (*p* < 1e-7) (Fig. 1a), whereas no significant change was found between paired samples collected from age-matched non-cancer female controls at two time points separated by a similar median of 130 days (IQR 92–155 days) (Fig. 1b). Adjusting for age at enrollment, the days between the two blood collections, or the days between the last infusion of chemotherapy drugs and the second blood collection had little impact on the results (data not shown).

**Fig. 2:**
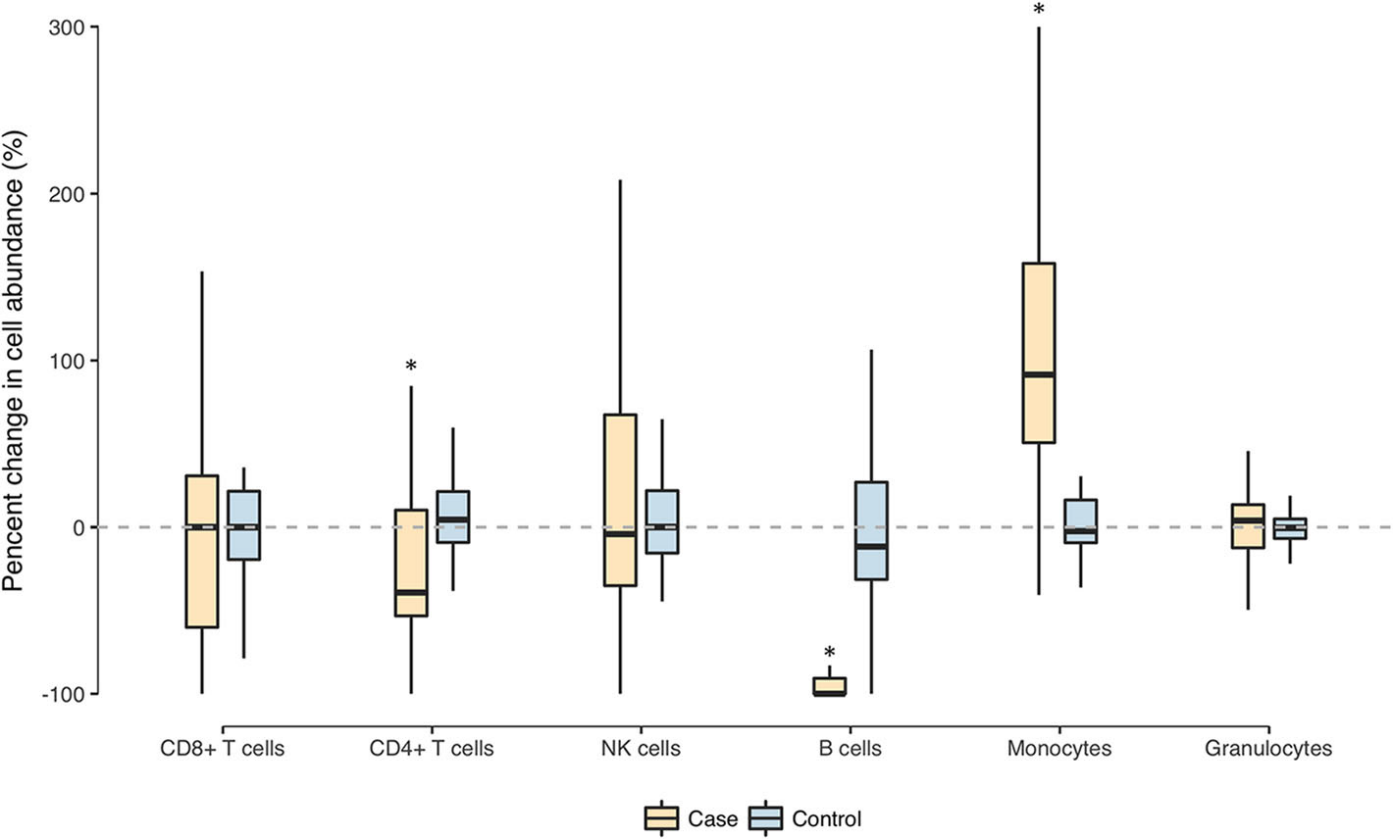
Change in the composition of leukocyte subtypes estimated from DNA methylation data in serial blood samples. The abundance of the six leukocyte subtypes was estimated as relative proportions based on DNA methylation at pre- and post-chemotherapy, and the percent change for each subtype was calculated between paired samples. The bar in the middle of the boxplot indicates the subgroup median, and the lower and upper edges indicate the first and third quartiles, respectively, for the percent change in the abundance of each leukocyte subtype. The dashed line indicates 0% change, and significant changes (different from zero), including CD4^+^ T cells, B cells, and monocytes in the case group, are marked by an asterisk above the box

Because of the heterogeneity in leukocyte composition of blood samples, the relative proportions of the six major leukocyte subtypes were estimated based on DNA methylation data and compared within paired samples. In breast cancer patients, the relative proportion of monocytes increased by a median of 91% (median absolute change 6.6%) after chemotherapy, whereas B cells and CD4-T cells decreased by a median of 100% (median absolute change − 2.5%) and 39% (median absolute change − 6.0%), respectively (all *p* values < 0.001) (Fig. 2). No significant change in leukocyte composition, however, was found in paired samples collected from non-cancer controls (Fig. 2).

We next performed a series of analyses to further characterize the associations of chemotherapy classification, regimen, dosage (i.e., cumulative infusions), and supportive treatment (growth factors and steroids) with the estimated leukocyte composition. As shown in Additional file 1: Figure S1A, the changes in leukocyte composition were similar between the adjuvant and the neoadjuvant settings, suggesting little impact of surgery prior to chemotherapy. The changes seen in the more detailed chemotherapy regimens (Additional file 1: Figure S1B) appeared to be driven mostly by anthracycline (Additional file 1: Figure S1C). Of the three leukocyte cell subtypes that were significantly altered by chemotherapy, the changes in the proportion of monocytes and B cells were consistent across those treatment subgroups, whereas the drop in CD4^+^ T cells was dependent on anthracycline and dose-dependent on cumulative dose of chemotherapy, growth factors, and steroids (Additional file 1: Figure S1). In addition, anthracycline treatment was associated with decreased CD8^+^ T cells, and anthracycline vs. non-anthracycline regimens appeared to have opposite effects on the proportion of granulocytes (Additional file 1: Figure S1C).

**Fig. 3:**
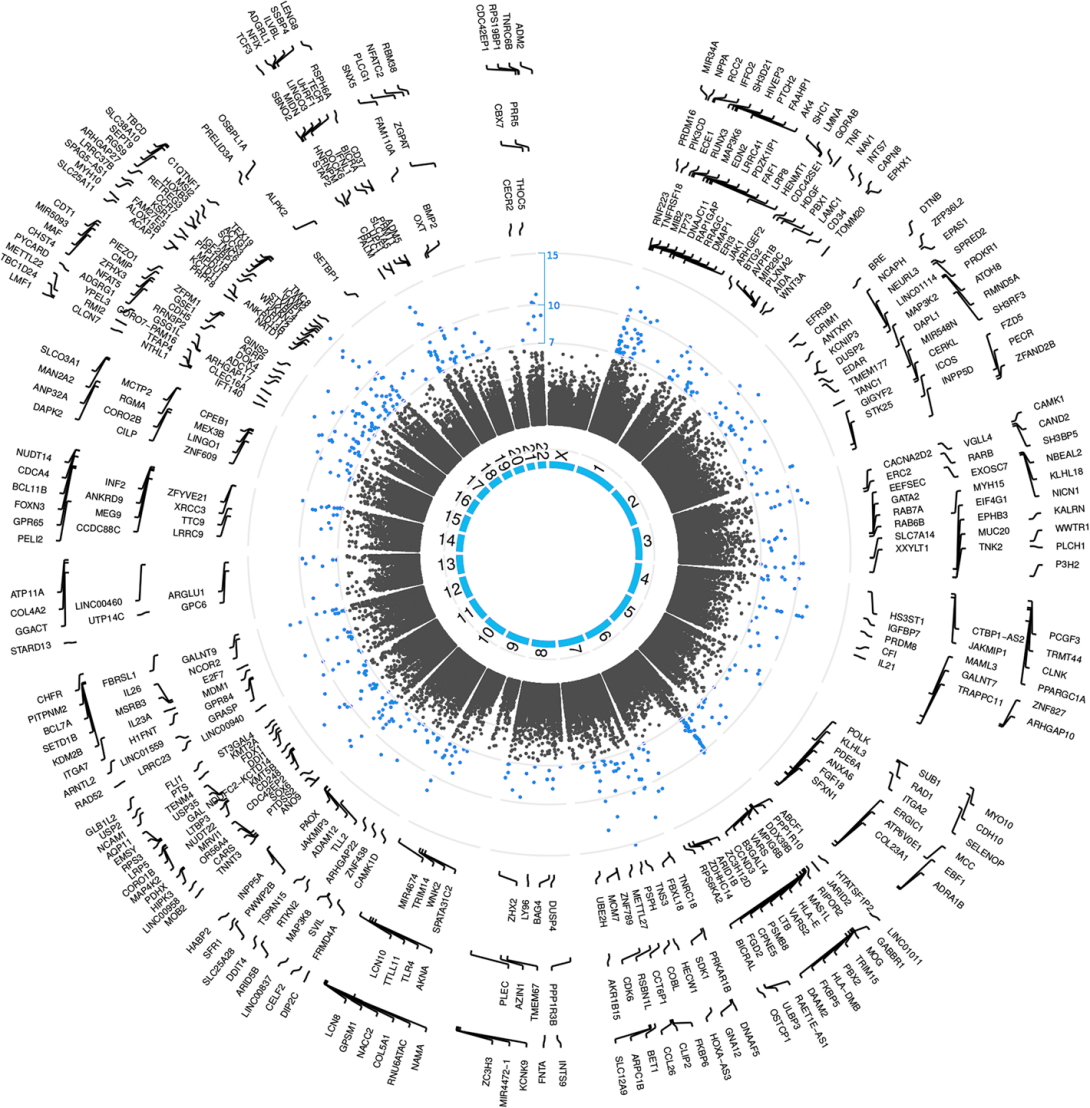
Circos plot of significant changes in blood DNA methylome before and after chemotherapy with adjustment for leukocyte composition. Leukocyte CpG sites that were significantly altered between pre- and post-chemotherapy after adjustment for leukocyte composition are displayed in circos plot. The inner most circle shows the 23 chromosomes. CpG sites organized based on chromosomal position and log-transformed *p* values are shown as closed dots, with significant CpG sites highlighted in blue (*p* < 1e-7 as shown in the red circular line). Symbols for the 460 genes annotated for those CpG sites are shown in the outer circles

After controlling for the estimated leukocyte composition in blood samples, while most of the previously observed methylation changes in the 16,679 CpG sites between pre- and post-chemotherapy became non-significant, 568 CpG probes from 460 genes in all but chromosomes 9, 21, and X remained significant (Fig. 3; summary statistics for all significant probes provided in Additional file 2: Table S2). Pathway enrichment analysis revealed signaling pathways regulating pluripotent stem cells as the most significantly enriched pathway, followed by pathways in cancer with a nominal *p* value ≤ 0.05 and a false discovery rate < 0.25 (Additional file 3: Table S3).

**Fig. 4:**
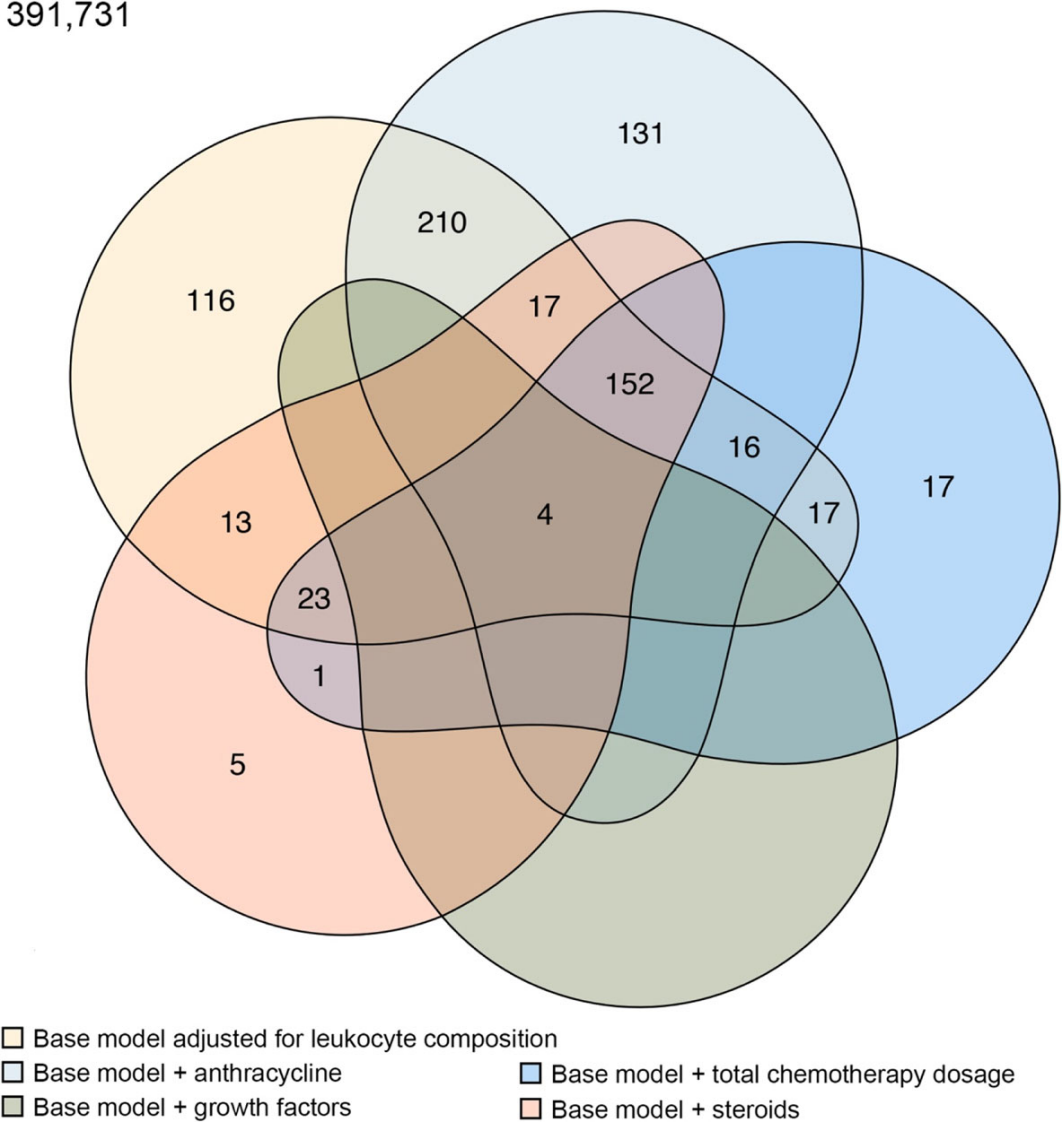
Venn diagram of significant CpG sites in multivariate models. The change of DNA methylation for each CpG site between pre- and post-chemotherapy was tested in linear regression models with adjustment for estimated leukocyte composition as the base model (base model), and in addition, for chemotherapy regimen (anthracycline vs. non-anthracycline), accumulative dose chemotherapeutic drugs, accumulative dose of growth factors administered, and accumulative dose of steroids administered. Significant CpG sites across the above five models are displayed in Venn diagram. Cutoff of significance level was set at 1e-7 for adjusting for approximately 500,000 tests

**Fig. 5:**
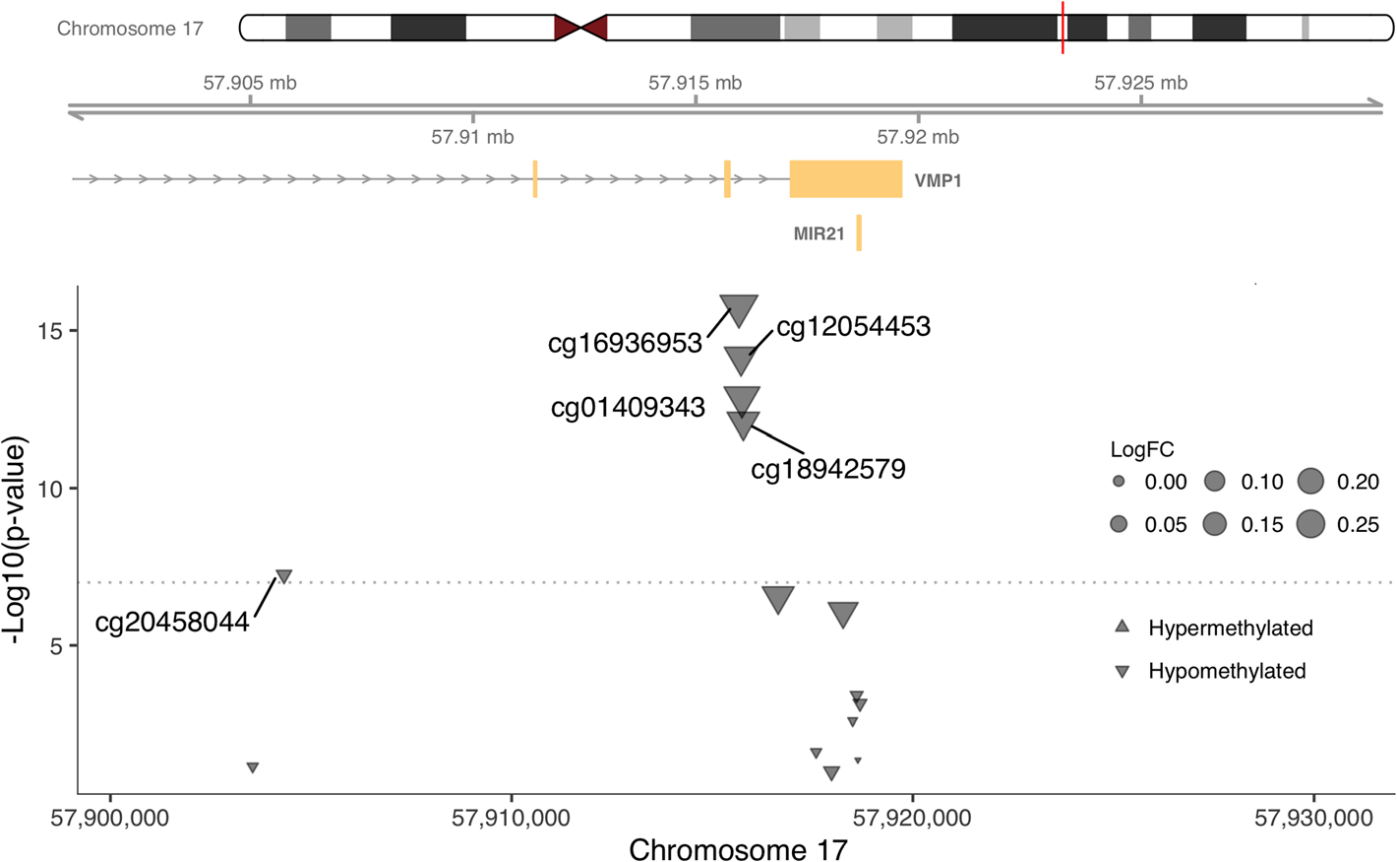
The VMP1/MIR21 locus where the DNA methylation status was most significantly altered after chemotherapy. The log10-transformed *p* values of DNA methylation changes between before and after chemotherapy with adjustment for cell composition are plotted against the chromosome locations of the CpG probes in the 500 kb region centering on cg16936953, the most significant probe. The size of a triangle indicates the log-transformed methylation level change (logFC) and the direction of a triangle indicates hypermethylation vs. hypomethylation. The chromosome ideogram and known genes in this locus are shown at the top of the plot

To test whether treatment modalities had any impact on the changes in methylation of the 568 identified CpG sites that were independent of leukocyte composition, each of the following factors, including chemotherapy regimens (anthracycline vs. non-anthracycline), cumulative infusion of chemotherapy, growth factors, and steroids, was added in turn to the base multivariable linear models containing leukocyte compositions. The overlap of significant CpGs from those models is shown in Fig. 4. Adjusting for the cumulative infusion of growth factors had the most drastic effects, with only four CpGs remaining significant in the models. These four CpGs, including cg16936953 in *VMP1*/*MIR21*, cg01252023 in *CORO1B*, cg11859398 in *SDK1*, and cg19956914 in *SUMF2*, were also the ones that were significant across all models (Table 1). CpG cg16936953, the most significant locus in both the unadjusted and adjusted models in our analysis (Fig. 5), was also the most significant locus in an earlier study comparing patients treated with chemotherapy and those untreated [4], as well as several epigenome-wide association studies (EWAS) of inflammation-related phenotypes [6–11]. CpG cg16936953 underwent significant hypomethylation after chemotherapy, a direction also consistent with the literature where the beta value of this CpG was negatively correlated with obesity and the levels of inflammation markers [6, 7] and lower in inflammation-related disease conditions [8–11].

**Table 1:** CpG probes that remain significant after adjustment for treatment modalities

CpG probe	logFC	*P* value	Chr	Position	RefGene_Name	RefGene_Group
cg16936953	0.262	1.71E-16	17	57,915,665	*VMP1*	gene body
cg19956914	− 0.093	4.56E-16	7	56,147,257	*SUMF2*	gene body
cg11859398	− 0.173	2.70E-14	7	3,411,503	*SDK1*	gene body
cg01252023	− 0.088	3.52E-14	11	67,207,574	*CORO1B*	gene body

**Fig. 6:**
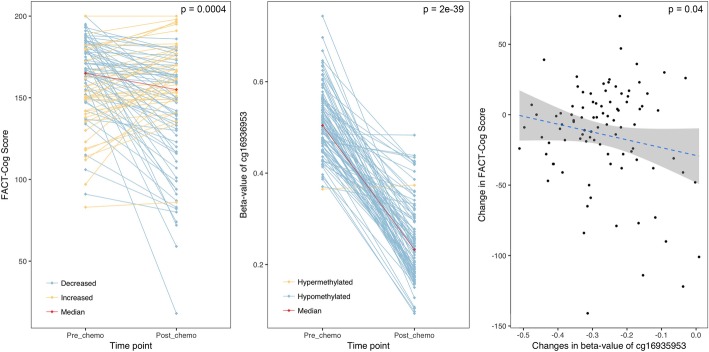
Changes in FACT-Cog score and CpG cg16936953 before and after chemotherapy. **a** FACT-Cog scores breast cancer patients at pre-chemotherapy and post-chemotherapy timepoints. Blue line indicates a decrease and yellow line indicates an increase in the score. Red line connects the median scores at the two timepoints. **b** Beta-values of CpG cg16936953 of breast cancer patients at pre-chemotherapy and post-chemotherapy timepoints. Blue line indicates a decrease and yellow line indicates an increase in the score. Red line connects the median scores at the two timepoints. **c** Correlation plot between the changes in FACT-Cog scores and the changes in beta-value of cg16936953 in breast cancer patients. The gray area around the blue regression line indicates 95% confidence intervals. Although data not shown, in cancer-free control, FACT-Cog scores were high at baseline, indicating no impairment, and did not change significantly over time

Lastly, we examined the above four CpGs with the decline of cognitive function after chemotherapy measured by FACT-Cog score. As shown in Fig. 6, a significant negative correlation was found between the change in FACT-Cog score and the change in methylation level of CpG cg16936953, but not in any of the other three CpGs.

## Discussion

It is widely speculated that chemotherapy has major effects on DNA methylation status of leukocytes, which may have implication on treatment responses and symptom outcomes; however, high-quality data from a properly designed study have been lacking. Using paired blood samples collected approximately 4 months apart at pre- and post-chemotherapy, we herein report for the first time an in-depth characterization of genome-wide methylation changes induced by chemotherapy in breast cancer patients. This was in stark contrast to the static methylome in untreated non-cancer controls. Given the relatively small sample size, it is rather remarkable that we could identify changes at more than 16,000 CpG sites at a genome-wide significance level, where 568 CpG sites remained significant after adjusting for leukocyte heterogeneity. This suggests that chemotherapy-induced DNA methylation changes in leukocytes were indeed widespread and profound.

Based on the epigenome-wide DNA methylation data, we provide a comprehensive profiling of the changes in leukocyte composition after chemotherapy by treatment setting (neoadjuvant vs. adjuvant), regimen, dose, and supportive therapy. The changes in leukocyte composition, including the decrease in B cells, CD4^+^ T cells, and CD8^+^ T cells (by anthracycline only) and the increase in monocytes, as estimated by DNA methylation profiles, are consistent with the findings from previous studies based on flow cytometry enumeration [12–16], which showed depletion of B cells and CD4^+^ T cells following chemotherapy and no recovery even at 9 months afterwards [12]. Moreover, our results revealed similar changes in leukocyte composition between the adjuvant and the neoadjuvant settings, and anthracycline was the chemotherapeutic agent driving most of the observed changes. Those changes in leukocytes likely reflect a combination of cytotoxicity to mature blood cells and bone marrow, disturbed hematopoietic lineaging, and various immune responses including inflammation induced by chemotherapy [17].

At the single CpG level, we identified four loci that remained significant across various models. The most significant CpG in our analysis, cg16936953, lies within a region containing *VMP1* and *MIR21*. In addition, another four neighboring CpG sites in this locus (cg12054453, cg01409343, cg18942579, and cg20458044) were similarly hypomethylated after chemotherapy (Fig. 5), all of which locate in or close to exon 11 that was identified as a promoter region on the primary transcript of *MIR21* [18]. Consistently, cg16936953 was also the most significant CpG that had a lower methylation level in patients treated with chemotherapy compared to those not treated in an earlier study [4]. Moreover, CpGs in the *VMP1*/*MIR21* locus are also among the top hits in several previous EWAS, including chronic inflammation marker C-reactive protein (CRP) [6], cardiovascular biomarker GDF-15 [7], obesity [8, 9], childhood-onset Crohn’s disease [10], and inflammatory bowel disease [11]. A lower methylation level of cg16936953 was invariably found in the above inflammation-related phenotypes or conditions. Thus, it is becoming evident that the hypomethylated status of *VMP1*/*MIR21* locus can potentially be used as a novel biomarker of systemic inflammation.

From a functional perspective, the hypomethylated *VMP1/MIR21* locus was associated with upregulated miR21 expression and a number of genes targeted by this microRNA in whole blood samples [18]. miR-21 is one the earliest characterized microRNAs in humans. Based on in silico prediction from miRWalk [19], as many as 3200 genes are likely under regulation by miR-21, making it one of the most versatile microRNAs regulating a myriad of biological processes, including various cancers and T cell differentiation and development [20]. Thus, it may not be surprising that MIR21 was the most responsive methylation locus to chemotherapeutic drugs. In recent years, there is a growing interest in the mechanistic, biomarker, and therapeutic roles of epigenetic pathways in response to chemotherapy [17, 21, 22]. The convergence of DNA methylation and microRNA as two potential epigenetic mechanisms on the same *VMP1*/*MIR21* region in response to chemotherapy is intriguing and warrants support for future research.

From a clinical perspective, it was recently shown that greater changes in leukocyte methylation tend to predict better treatment outcomes by platinum-based chemotherapy for ovarian cancer, and some of the leukocyte methylation changes mirrored those in tumor tissues at relapse [3]. The cross-tissue preservation of epigenetic response to chemotherapy is particularly intriguing, indicating the potential use of peripheral blood to monitor treatment response in tumors. Future studies are warranted to determine the longitudinal relationships among leukocyte methylation changes, inflammation and other immune responses, and patient outcomes and symptoms after chemotherapy. We also plan to validate the finding of cg16936953 in the *VMP1*/*MIR21* region with cognitive function in a larger longitudinal study assessing subjective and objectively measured cognitive function. Some recent animal studies show the involvement of miR-21 in cognitive improvement following exercise and in cognitive behavior after spared nerve surgery [23, 24]. However, to date, we still know little about microRNAs and cognitive behaviors, or whether leukocyte methylation regulation as measured in our study is conserved in the central nervous system given tissue specificity of epigenetic regulations. Further elucidation of the epigenetic pathways that could be involved in the association we identified and cross-tissue validation are warranted.

Our study is unique in its design of paired sample collection pre- and post-chemotherapy and the availability of paired samples from untreated, non-cancer patients as a control group. Additionally, the findings are generalizable based on the nationwide study. Because of the relatively small sample size, the statistical power to relate the methylation changes with clinical endpoints, including cognitive function, was limited. Another limitation is the lack of sample collection at multiple later timepoints post-chemotherapy. It was reported that the populations of B cells and CD4^+^ T cells did not recover even at 9 months after treatment [12]. It will be interesting to study the impact of chemotherapy on leukocyte profiles and DNA methylation status at long-term to assess whether some of the changes are perpetuated on the epigenome as a part of cellular memory. Lastly, it remains to be determined the impact of the changes in DNA methylation of the identified loci on the expression level of related genes after chemotherapy, which will provide important functional and mechanistic insights into the systemic response to chemotherapy through epigenetic regulation.

In summary, we show that chemotherapy profoundly alters the DNA methylation landscape of leukocytes in breast cancer patients, in contrast to the stable methylome in non-treated controls. Our results shed light on the epigenetic response of circulating immune cell populations to cytotoxic chemotherapeutic drugs. The correlation between methylation changes in cg16936953 and cognitive function suggests that there is a potential for using blood methylation as non-invasive markers to predict treatment response and symptom outcomes.

## Methods

### Study design, measures, and participants

As previously described, breast cancer patients and healthy non-cancer controls were recruited from the National Cancer Institute (NCI) Community Oncology Research Program (NCORP) locations nationwide to participate in a study investigating the trajectory of changes in cognitive function before and following chemotherapy compared to controls assessed at the same times [5]. Breast cancer participant eligibility included (1) female with stage I–IIIC disease, (2) scheduled for a standard course of chemotherapy (adjuvant or neoadjuvant), (3) chemotherapy naïve, (4) 21 years of age or older, (5) no CNS disease, (6) never diagnosed with a neurodegenerative disease, (7) no recent major psychiatric illness, and (8) no plan to receive concurrent radiation from pre- to post-chemotherapy. Control participants were the same age (within 5 years) as the paired breast cancer participants and met eligibility criteria 3–7. Relevant clinical and demographic information was obtained from the medical record and patient questionnaire, respectively. Perceived cognitive function was assessed at the clinic location by using the Functional Assessment of Cancer Therapy-Cognitive Function (FACT-Cog), version 2, a well-validated questionnaire to address perceived cancer-related cognitive impairment [25]. This study was approved by the Institutional Review Board of each NCORP, the University of Rochester Cancer Center (URCC) NCORP Research Base, and Roswell Park Comprehensive Cancer Center; all participants provided informed consent.

### Procurement of blood samples

All blood samples were collected at NCORP sites and shipped to the Cancer Control and Psychoneuroimmunology Lab (CCPL) at the University of Rochester Cancer Center NCORP Research Base. Whole blood was drawn in vacutainers with EDTA and frozen at − 80 °C or − 20 °C prior to storing at − 80 °C. At the CCPL, all samples were stored at − 80 °C prior to shipping them to Roswell Park Data Bank and BioRepoistory (DBBR) laboratories for DNA extraction using Qiagen QIAamp DNA Blood mini kit and quantification by NanoDrop and Qubit technologies. This study included paired pre- and post-chemotherapy samples from 93 breast cancer cases with a median of 128 days apart (range 71–230 days; interquartile range [IQR] 113–157 days), as well as 48 non-cancer controls assessed at the same times as cases with a median of 130 days apart (range 42–453 days; IQR 92–155 days). Descriptive characteristics of the case and control groups are provided in Additional file 4: Table S1.

### DNA methylation array assays and data processing

To minimize potential batch effects, DNA samples were randomized to batches of 12 based on key variables including case-control status, time of collection, and age at enrollment using Bioconductor package *OSAT* [26]. For each sample, 1 μg of DNA was aliquoted and subjected to bisulfite conversion using the Zymo EZ DNA Methylation Kit, and 500 ng of converted DNA was used as input for the DNA methylation microarray assay using Illumina Infinium HumanMethylation450 Beadchip. The assays were performed by Roswell Park Genomics Shared Resource (GSR) laboratory following the manufacturer’s protocol. The raw data were processed by the R package “minfi” [27], and converted to methylation ß-value, ranging from 0 to 1 with 0 being unmethylated and 1 being fully methylated, to represent the methylation level of each CpG site. Methylation data were normalized by the *SWAN* method implemented in *minfi* package [28] to correct for technical bias due to the use of two types of probe chemistry, and residual batch effects were further corrected using the *ComBat* program [29]. Problematic probes and samples with poor detection *p* values were removed using the R package *IMA* [30], which resulted in the removal of 3 cases and 93,059 CpG probes, including those close to SNPs in the probe sequence. The final dataset included 392,453 CpG probes from paired samples from 93 cases and 48 controls.

### Data analysis

Changes in ß-value between pre- and post-treatment blood samples for cases or between the two consecutive time points for controls were tested using the linear model as implemented in the *limma* method of *minfi* package [27]. Multiple testing was corrected using a stringent Bonferroni-adjusted threshold of 1e-7. To investigate leukocyte composition and the changes between the two blood collections, a reference-based deconvolution method by Houseman et al. was applied [31], which estimates the relative proportion of six major leukocyte subtypes, including granulocytes, monocytes, CD4^+^ T cells, CD8^+^ T cells, B cells, and natural killer (NK) cells, adding up to 100%. Changes in each leukocyte cell type between the two blood collections were examined using paired *t* tests in cases and controls separately, as well as in cases stratified by treatment factors including chemotherapy setting, regimen, dosage, and supportive treatment (grow factors and steroids). To investigate whether the observed DNA methylation changes following chemotherapy were due to altered leukocyte composition in whole blood samples, changes in the estimated leukocyte proportions were adjusted in *limma* models described above. Further adjustment for treatment factors was also performed in the *limma*-based linear model. Gene set enrichment analysis was performed using DAVID online tool with default setting [32].

## Additional files

Additional file 1: **Figure S1**. Changes in leukocyte composition before and after chemotherapy by treatment regimen, growth factor and steroid use. The abundance of the 6 leukocyte subtypes was estimated as relative proportions based on DNA methylation at pre- and post-chemotherapy, and the percent change for each subtype was calculated between paired samples. The bar in the middle of the boxplot indicates the subgroup median, and the lower and upper edges indicate the first and third quartiles, respectively, for the percent change in the abundance of each leukocyte subtype. The dashed line indicates 0 % change. Panels A-F are: A) chemotherapy setting; B) chemotherapy regimen (“other” group all had adriamycin); C) anthracycline; D) total chemotherapy infusion; E) total growth factor infusion; F) total steroid infusion. (DOCX 307 kb)

Additional file 2: **Table S2.** Summary statistics of the 568 CpGs which remain significantly altered by chemotherapy after adjustment for leukocyte composition. (PDF 282 kb)

Additional file 3: **Table S3.** Pathway enrichment analysis of significant CpG sites altered between pre- and post-chemotherapy with adjustment for leukocyte composition (DOCX 15 kb)

Additional file 4: **Table S1.** Descriptive characteristics of breast cancer cases and non-cancer controls. (DOCX 17 kb)

## Abbreviations

CCPL: Cancer Control and Psychoneuroimmunology Lab
CRP: C-reactive protein
DBBR: Data Bank and BioRepository
IQR: Interquartile range
NCI: National Cancer Institute
NCORP: NCI Community Oncology Research Program
NK: Natural killer
