# Supplementary material for: Impact of chemotherapy for breast cancer on leukocyte DNA methylation landscape and cognitive function: a prospective study
Source: Clin Epigenetics. 2019 Mar 12;11:45. doi: 10.1186/s13148-019-0641-1 (PMC6416954; doi:10.1186/s13148-019-0641-1)

**Supplemental Figure S1.** Changes in leukocyte composition before and after chemotherapy by treatment regimen, growth factor and steroid use


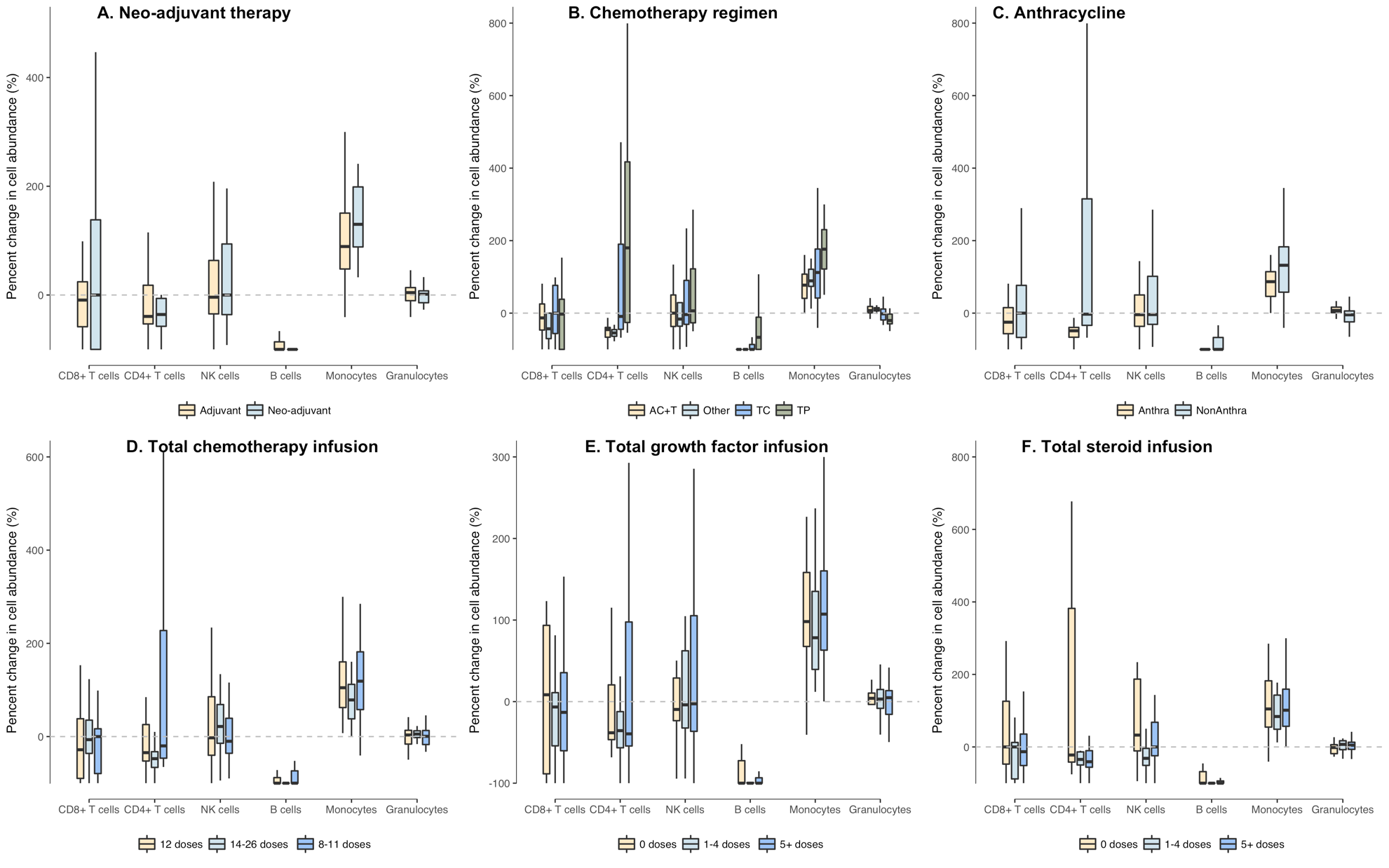

Supplement: Supplementary file 1 — Figure S1. Changes in leukocyte composition before and after chemotherapy by treatment regimen, growth factor and steroid use. The abundance of the 6 leukocyte subtypes was estimated as relative proportions based on DNA methylation at pre- and post-chemotherapy, and the percent change for each subtype was calculated between paired samples. The bar in the middle of the boxplot indicates the subgroup median, and the lower and upper edges indicate the first and third quartiles, respectively, for the percent change in the abundance of each leukocyte subtype. The dashed line indicates 0 % change. Panels A-F are: A) chemotherapy setting; B) chemotherapy regimen (“other” group all had adriamycin); C) anthracycline; D) total chemotherapy infusion; E) total growth factor infusion; F) total steroid infusion. (DOCX 307 kb) [file 13148_2019_641_MOESM1_ESM.docx]
