# Supplementary material for: Impact of chemotherapy for breast cancer on leukocyte DNA methylation landscape and cognitive function: a prospective study
Source: Clin Epigenetics. 2019 Mar 12;11:45. doi: 10.1186/s13148-019-0641-1 (PMC6416954; doi:10.1186/s13148-019-0641-1)
Supplement: Supplementary file 2 — Table S2. Summary statistics of the 568 CpGs which remain significantly altered by chemotherapy after adjustment for leukocyte composition. (PDF 282 kb) [file 13148_2019_641_MOESM2_ESM.pdf]

ILMNID	logFC	AveExpr	P.Value	GENOME_BU	CHR	MAPINFO	UCSC_REFGE	UCSC_REFGE	UCSC_REFGENE_GROU	UCSC_CPG_I	RELATION_T	PHANTOM	DMR	ENHANCER	HMM_ISLAN	REGULATOR	REGULATOR	DHS	Index
cg16936953	-0.2622273	0.2673594	1.71E-16	37	17	57915665	TMEM49	NM_030938	Body	NA	NA	low-CpG:552	NA	NA	NA	NA	NA	NA	310947
cg19956914	0.09252384	-0.0671882	4.56E-16	37	7	56147257	SUMF2;SUM	NM_001042	Body;Body;Body;Body;	NA	NA	NA	NA	TRUE	NA	7:56146865-	Gene_Associ	TRUE	359545
cg12054453	-0.2001315	0.2240511	6.95E-15	37	17	57915717	TMEM49	NM_030938	Body	NA	NA	low-CpG:552	NA	NA	NA	NA	NA	NA	225475
cg06827038	0.05004178	-0.0413071	1.69E-14	37	17	48912952	WFIKKN2;W	NM_175575;	1stExon;5'UTR	NA	NA	NA	NA	NA	17:46267446	NA	NA	NA	133233
cg08173263	0.25841685	-0.2560454	2.67E-14	37	19	14276911	LPHN1;LPHN	NM_014921;	Body;Body	chr19:14273	S_Shelf	NA	NA	TRUE	NA	19:14276822	Unclassified_	NA	158664
cg11859398	0.17279342	-0.1170906	2.70E-14	37	7	3411503	SDK1	NM_152744	Body	NA	NA	NA	DMR	TRUE	NA	NA	NA	NA	222277
cg01252023	0.08824122	-0.0675643	3.52E-14	37	11	67207574	CORO1B;CO	NM_001018	Body;Body	chr11:67203	S_Shelf	NA	NA	NA	11:66964001	NA	NA	NA	26238
cg05715492	0.1123228	-0.0882284	3.63E-14	37	7	98991138	ARPC1B	NM_005720	Body	chr7:989901	S_Shore	NA	CDMR	TRUE	7:98828146-	7:98990227-	Promoter_As	TRUE	112976
cg17445936	0.07149699	-0.0468052	4.57E-14	37	1	3090345	PRDM16;PRD	NM_022114;	Body;Body	NA	NA	NA	NA	NA	1:3079749-3	NA	NA	NA	319165
cg23973524	0.09588504	-0.0925909	1.18E-13	37	19	18873222	CRTC1;CRTC1	NM_015321;	Body;Body	chr19:18873	Island	NA	NA	NA	19:18734020	NA	NA	TRUE	421430
cg24420089	0.10064794	-0.081808	1.34E-13	37	11	457304	PTDSS2	NM_030783	Body	chr11:45908	N_Shore	NA	NA	NA	11:447256-4	11:456465-4	Unclassified_	NA	428664
cg01409343	-0.2328717	0.21861979	1.34E-13	37	17	57915740	TMEM49	NM_030938	Body	NA	NA	low-CpG:552	NA	NA	NA	NA	NA	NA	29286
cg06647693	0.12822382	-0.1022288	1.66E-13	37	16	88590340	ZFPM1	NM_153813	Body	chr16:88590	Island	NA	NA	NA	16:87117783	NA	NA	NA	129668
cg15283950	0.09780537	-0.0805671	2.49E-13	37	11	10715217	MRVI1;MRV	NM_001100	1stExon;5'UTR;1stExon	NA	NA	low-CpG:106	NA	TRUE	NA	NA	NA	NA	283209
cg26709300	0.10241641	-0.1122991	2.89E-13	37	16	30106682	YPEL3;YPEL3	NM_031477;	1stExon;Body	chr16:30107	N_Shore	NA	NA	TRUE	16:30014183	NA	NA	NA	466075
cg11303839	-0.2157546	0.22230688	3.79E-13	37	7	75405967	CCL26	NM_006072	5'UTR	NA	NA	NA	NA	NA	NA	7:75405958-	Unclassified_	NA	212943
cg24693803	0.07362217	-0.0705283	5.59E-13	37	4	171013537	NA	NA	NA	chr4:171010	S_Shelf	NA	NA	NA	4:171250051	NA	NA	NA	433238
cg21867733	0.07485671	-0.0691131	6.54E-13	37	12	124985584	NCOR2;NCO	NM_006312;	5'UTR;5'UTR	NA	NA	NA	NA	TRUE	12:12355137	NA	NA	TRUE	388475
cg18942579	-0.187228	0.20011295	7.92E-13	37	17	57915773	TMEM49	NM_030938	Body	NA	NA	low-CpG:552	NA	NA	NA	17:57915757	Promoter_As	NA	343287
cg13485366	0.09358523	-0.0872452	1.14E-12	37	9	137174989	NA	NA	NA	NA	NA	NA	NA	TRUE	NA	NA	NA	TRUE	248731
cg24277817	0.09648754	-0.0812236	1.38E-12	37	19	13135662	NFIX	NM_002501	Body	chr19:13135	Island	low-CpG:129	NA	NA	19:12996280	NA	NA	NA	426383
cg09938049	0.06552559	-0.0652706	1.54E-12	37	12	54747206	NA	NA	NA	NA	NA	NA	NA	TRUE	NA	NA	NA	NA	189632
cg14242895	0.10407159	-0.086074	1.55E-12	37	11	118393450	MLL	NM_005933	3'UTR	NA	NA	NA	NA	NA	NA	11:11839317	Gene_Associ	NA	264546
cg23989207	0.08397289	-0.0442871	1.83E-12	37	17	80870107	TBCD	NM_005993	Body	chr17:80871	N_Shore	NA	NA	NA	17:78463396	NA	NA	NA	421723
cg01751245	-0.1972889	0.20433263	1.91E-12	37	2	65593761	SPRED2;SPR	NM_001128	5'UTR;1stExon;Body	NA	NA	NA	NA	TRUE	NA	NA	NA	TRUE	35835
cg10753966	0.09968469	-0.1033172	1.95E-12	37	3	55556197	ERC2	NM_015576	3'UTR	NA	NA	NA	NA	TRUE	NA	NA	NA	NA	203729
cg21154227	0.07317468	-0.0501941	2.17E-12	37	4	57904332	IGFBP7	NM_001553	Body	NA	NA	NA	NA	TRUE	NA	4:57903701-	Unclassified_	TRUE	377635
cg24878115	0.12963311	-0.1137889	2.26E-12	37	19	18545062	SSBP4;SSBP4	NM_032627;	3'UTR;3'UTR	chr19:18543	Island	NA	NA	NA	19:18404916	19:18543757	Unclassified_	NA	436405
cg10549018	0.10598415	-0.0909524	2.65E-12	37	10	98176740	TLL2	NM_012465	Body	NA	NA	NA	NA	TRUE	NA	NA	NA	NA	200415
cg22761205	0.07880295	-0.070848	2.76E-12	37	11	457256	PTDSS2	NM_030783	Body	chr11:45908	N_Shore	NA	NA	NA	11:447256-4	11:456465-4	Unclassified_	NA	401597
cg17501210	-0.1533787	0.17673271	3.30E-12	37	6	166970252	RPS6KA2;RP	NM_021135;	Body;Body	NA	NA	NA	RDMR	NA	NA	NA	NA	TRUE	320131
cg22902505	0.13262803	-0.1111155	3.47E-12	37	4	81119473	PRDM8;PRD	NM_020226;	5'UTR;5'UTR	chr4:811181	S_Shore	NA	NA	NA	4:81338101-	NA	NA	NA	404009
cg22778120	0.08590531	-0.0663042	3.48E-12	37	22	45094531	PRR5;PRR5;P	NM_001017	5'UTR;5'UTR;5'UTR;5'	chr22:45097	N_Shelf	NA	RDMR	TRUE	NA	NA	NA	TRUE	401831
cg06307913	0.17945762	-0.1641137	3.51E-12	37	4	81118794	PRDM8;PRD	NM_020226;	5'UTR;1stExon;5'UTR	chr4:811190	N_Shore	NA	NA	NA	NA	4:81118358-	Unclassified_	NA	123528
cg03561416	0.0553215	-0.0487209	3.78E-12	37	6	157254584	ARID1B;ARID	NM_017519;	Body;Body;Body	NA	NA	NA	NA	TRUE	NA	NA	NA	NA	72040
cg02023728	0.13788256	-0.1058241	4.16E-12	37	11	77925099	USP35	NM_020798	3'UTR	chr11:77921	S_Shelf	NA	NA	TRUE	NA	NA	NA	NA	41511
cg03829194	0.08749342	-0.068622	4.30E-12	37	16	875571	NA	NA	NA	chr16:87510	Island	NA	NA	TRUE	16:814282-8	NA	NA	TRUE	77198
cg22153728	0.07634917	-0.065301	4.34E-12	37	1	25291584	RUNX3	NM_001031	TSS200	NA	NA	NA	NA	NA	1:25163972-	1:25291322-	Unclassified	NA	392686
cg21993290	-0.0685025	0.0692106	4.87E-12	37	2	233703120	GIGYF2;GIGY	NM_015575;	Body;Body;Body;Body	NA	NA	NA	NA	TRUE	NA	2:233703065	Gene_Associ	NA	390303
cg09109520	0.11386229	-0.122112	5.19E-12	37	16	57673258	GPR56;GPR5	NM_001145	5'UTR;5'UTR;5'UTR;5'	NA	NA	NA	NA	NA	NA	NA	NA	NA	175192
cg11639950	0.05204619	-0.0160348	5.69E-12	37	12	125003407	NCOR2;NCO	NM_006312;	5'UTR;5'UTR	chr12:12500	Island	NA	RDMR	TRUE	12:12356873	NA	NA	NA	218232
cg26432350	0.10412858	-0.0985446	5.87E-12	37	3	47040357	NBEAL2	NM_015175	Body	NA	NA	NA	NA	NA	3:47015218-	3:47039622-	Gene_Associ	NA	461266
cg10493186	0.12088323	-0.100672	6.29E-12	37	1	3134756	PRDM16;PRD	NM_022114;	Body;Body	NA	NA	NA	NA	NA	1:3123982-3	NA	NA	NA	199262
cg00153395	0.06640361	-0.0517784	6.72E-12	37	1	65327523	JAK1	NM_002227	Body	NA	NA	NA	NA	TRUE	NA	1:65327163-	Unclassified_	NA	3440
cg00460436	0.06063667	-0.039384	7.48E-12	37	16	4401729	Magmas	NM_016069	TSS1500	chr16:44009	S_Shore	NA	NA	NA	NA	16:4400653-	Promoter_As	NA	9726
cg03463411	0.13319831	-0.1159325	7.53E-12	37	4	81118188	PRDM8;PRD	NM_001099	TSS1500;5'UTR	chr4:811181	Island	NA	NA	NA	4:81336961-	NA	NA	NA	70030
cg27242132	0.15782078	-0.1265964	7.79E-12	37	4	81119178	PRDM8;PRD	NM_020226;	5'UTR;5'UTR	chr4:811190	Island	NA	NA	NA	4:81338101-	4:81118358-	Unclassified_	NA	475212
cg21646032	-0.0943478	0.10865854	8.23E-12	37	3	25623905	RARB;RARB	NM_016152;	Body;Body	NA	NA	NA	NA	TRUE	NA	NA	NA	TRUE	385323
cg25953130	-0.1646124	0.15790135	8.26E-12	37	10	63753550	ARID5B	NM_032199	Body	NA	NA	NA	NA	TRUE	NA	NA	NA	NA	453012
cg05208178	0.03885806	-0.0405	8.47E-12	37	2	233928423	INPP5D;INPP	NM_005541;	Body;Body	chr2:233925	S_Shelf	NA	DMR	NA	NA	2:233925718	Promoter_As	NA	103599
cg26282236	0.11268744	-0.1100402	8.86E-12	37	12	1025755	RAD52	NM_134424	Body	NA	NA	NA	NA	NA	12:895636-8	NA	NA	NA	458588
cg14145338	0.08724819	-0.0717372	1.03E-11	37	9	139649039	LCN8	NM_178469	Body	NA	NA	NA	NA	NA	9:138768786	NA	NA	NA	262385
cg11848788	-0.0684347	0.04902476	1.04E-11	37	1	39041399	NA	NA	NA	chr1:390440	N_Shelf	NA	NA	NA	NA	1:39041348-	Unclassified_	NA	222051
cg26299084	0.13171833	-0.1111933	1.16E-11	37	4	81118588	PRDM8;PRD	NM_020226;	5'UTR;TSS200	chr4:811181	Island	NA	NA	NA	4:81336961-	4:81118358-	Unclassified_	NA	458882
cg25446789	-0.1058468	0.12138812	1.18E-11	37	2	25810393	DTNB;DTNB;	NM_183361;	Body;Body;Body;Body;	NA	NA	NA	NA	TRUE	NA	2:25810375-	Unclassified_	NA	445348
cg21183455	0.06802056	-0.0483173	1.20E-11	37	22	50052396	C22orf34	NR_026997	TSS1500	NA	NA	NA	NA	NA	NA	22:50052284	Unclassified_	NA	378196
cg07435331	0.07172456	-0.0388404	1.29E-11	37	17	21178476	NA	NA	NA	chr17:21178	N_Shore	NA	NA	NA	NA	NA	NA	NA	145046
cg04509882	0.07997716	-0.0524453	1.32E-11	37	3	184038317	EIF4G1;EIF4	NM_198241;	Body;Body;Body;Body;1	NA	NA	low-CpG:185	NA	NA	NA	NA	NA	TRUE	90368
cg11261850	0.09481032	-0.0833498	1.38E-11	37	11	76320970	NA	NA	NA	NA	NA	NA	NA	TRUE	NA	NA	NA	NA	212269
cg20460227	0.06999472	-0.0466908	1.46E-11	37	2	120452632	NA	NA	NA	NA	NA	NA	NA	TRUE	NA	2:120452553	Unclassified_	TRUE	367073
cg20507228	0.15697509	-0.1483379	1.48E-11	37	15	91460071	MAN2A2	NM_006122	Body	NA	NA	NA	NA	TRUE	NA	15:91459961	Promoter_As	NA	367793
cg11950754	0.07809736	-0.065873	1.56E-11	37	1	53782077	LRP8;LRP8;L	NM_033300;	Body;Body;Body;Body	NA	NA	NA	NA	TRUE	NA	NA	NA	NA	223772
cg10420527	0.07923189	-0.0472183	1.57E-11	37	11	68138505	LRP5	NM_002335	Body	NA	NA	NA	CDMR	NA	NA	11:68138106	Unclassified_	NA	198036
cg06688763	0.1371532	-0.1403413	1.59E-11	37	7	92238207	CDK6;CDK6	NM_001259;	3'UTR;3'UTR	NA	NA	NA	CDMR	NA	7:92076022-	NA	NA	NA	130414
cg13694927	0.07129561	-0.0448542	1.64E-11	37	7	27184712	HOXA5	NM_019102	TSS1500	chr7:271826	Island	NA	NA	NA	7:27150952-	NA	NA	NA	253175
cg25318189	0.05292887	-0.029413	1.75E-11	37	7	2800436	GNA12	NM_007353	Body	chr7:280244	N_Shelf	NA	NA	TRUE	NA	7:2799907-2	Unclassified_	TRUE	443246

cg27115863	-0.1439506	0.16595535	1.81E-11	37	22	37921640	NA	NA	NA	NA	NA	NA	DMR	TRUE	NA	NA	NA	TRUE	473147
cg12798040	0.12793821	-0.1279304	1.95E-11	37	14	104171840	XRCC3;XRCC	NM_00110	Body;Body;Body	NA	NA	NA	NA	NA	14:10324136	NA	NA	NA	237366
cg14438453	0.12760313	-0.0851365	2.08E-11	37	6	39786566	DAAM2	NM_015345	5'UTR	NA	NA	NA	NA	TRUE	NA	NA	NA	NA	268454
cg27093918	-0.0874793	0.09616544	2.14E-11	37	16	69564625	NA	NA	NA	NA	NA	NA	DMR	TRUE	NA	16:69564532	Promoter_As	TRUE	472772
cg23605961	0.11012014	-0.0553074	2.18E-11	37	7	751331	PRKAR1B;PR	NM_002735;	5'UTR;5'UTR;5'UTR;5'	chr7:751712-	N_Shore	NA	NA	NA	7:717343-71	NA	NA	TRUE	415501
cg22508831	-0.1062662	0.10161154	2.41E-11	37	22	40573204	TNRC6B;TNR	NM_001024	Body;TSS1500;TSS150	NA	NA	NA	NA	NA	NA	22:40573013	Promoter_As	NA	397733
cg17417856	-0.1277042	0.12097025	2.48E-11	37	19	50191637	PRMT1;C19o	NM_001536;	3'UTR;TSS1500;3'UTR;	chr19:50192	N_Shore	NA	NA	NA	NA	NA	NA	NA	318711
cg25214914	0.05637796	-0.0288136	2.59E-11	37	1	155910523	RXFP4	NM_181885	TSS1500	NA	NA	NA	NA	NA	NA	1:155910341	Unclassified_	NA	441730
cg10843276	0.11892337	-0.1170302	2.63E-11	37	4	726697	PCGF3	NM_006315	5'UTR	chr4:724335-	S_Shore	NA	NA	NA	4:716385-71	NA	NA	NA	205367
cg08822118	0.07658383	-0.0528464	2.65E-11	37	3	184037481	EIF4G1;EIF4	NM_198241;	Body;Body;TSS1500;5'	NA	NA	low-CpG:185	NA	TRUE	NA	NA	NA	TRUE	170178
cg01418153	0.08648146	-0.0637685	2.80E-11	37	1	3240129	PRDM16;PRD	NM_022114;	Body;Body	chr1:323991	Island	NA	NA	NA	1:3229442-3	NA	NA	NA	29492
cg26165146	-0.1208445	0.13059873	2.92E-11	37	12	27484656	ARNTL2	NM_020183	TSS1500	chr12:27485	N_Shore	NA	RDMR	NA	NA	NA	NA	TRUE	456598
cg06378491	0.05909535	-0.046562	3.13E-11	37	11	64564012	MAP4K2	NM_004579	Body	NA	NA	NA	NA	NA	NA	NA	NA	NA	124748
cg18181703	-0.1101846	0.11115366	3.14E-11	37	17	76354621	SOCS3	NM_003955	Body	chr17:76354	N_Shore	NA	NA	NA	17:73866128	17:76353631	Promoter_As	NA	330978
cg22878489	0.1012436	-0.0900032	3.40E-11	37	6	33245701	B3GALT4	NM_003782	1stExon	chr6:332446	S_Shore	NA	NA	TRUE	6:33352687-	6:33244505-	Promoter_As	TRUE	403631
cg14029001	0.0764577	-0.0623119	3.84E-11	37	6	41998500	CCND3;CCND	NM_00113	5'UTR;5'UTR	NA	NA	NA	NA	TRUE	NA	NA	NA	NA	260028
cg01154283	0.09575413	-0.0565443	3.93E-11	37	2	36603543	CRIM1	NM_016441	Body	NA	NA	NA	NA	TRUE	NA	NA	NA	NA	24310
cg19309676	-0.1108898	0.09708504	4.15E-11	37	19	50191439	C19orf76;PR	NM_001101	TSS1500;Body;Body;Bo	chr19:50191	Island	NA	NA	NA	NA	NA	NA	NA	348671
cg26105278	-0.0460512	0.03699044	4.16E-11	37	1	3624054	TP73;TP73;T	NM_001126	Body;Body;Body;Body	chr1:362331	S_Shore	NA	NA	NA	1:3613683-3	NA	NA	TRUE	455457
cg18096987	0.06753141	-0.0480959	4.35E-11	37	3	11623873	VGLL4;VGLL	NM_014667;	Body;TSS200;Body	NA	NA	NA	NA	TRUE	NA	3:11623723-	Unclassified_	TRUE	329387
cg00317577	0.07364932	-0.0669791	4.78E-11	37	15	83239908	CPEB1;CPEB1	NM_001079	Body;Body;Body;Body	NA	NA	NA	NA	TRUE	NA	NA	NA	NA	6671
cg18099096	0.09146153	-0.0627509	4.91E-11	37	4	23936764	NA	NA	NA	NA	NA	NA	NA	TRUE	NA	NA	NA	NA	329432
cg27333952	0.08385479	-0.0741955	4.93E-11	37	16	1509206	CLCN7;CLCN	NM_001287;	Body;Body	chr16:15108	N_Shore	NA	NA	NA	16:1449098-	NA	NA	NA	476858
cg25310824	0.13768541	-0.0889545	5.34E-11	37	5	42812334	SEPP1;SEPP1	NM_005410;	TSS1500;TSS1500;TSS1	NA	NA	NA	NA	NA	NA	NA	NA	NA	443117
cg04543901	-0.1036169	0.07920682	5.41E-11	37	17	63519783	NA	NA	NA	NA	NA	NA	NA	TRUE	NA	17:63519568	Unclassified_	NA	90997
cg01109535	-0.0591311	0.04850748	5.59E-11	37	16	24856104	SLC5A11	NM_052944	TSS1500	NA	NA	NA	NA	NA	NA	NA	NA	TRUE	23434
cg01631333	0.07130688	-0.051179	5.78E-11	37	13	110747452	NA	NA	NA	NA	NA	NA	NA	TRUE	NA	NA	NA	TRUE	33717
cg21848084	0.07965028	-0.0793289	6.22E-11	37	1	3264381	PRDM16;PRD	NM_022114;	Body;Body	NA	NA	NA	NA	NA	NA	1:3264343-3	Unclassified_	NA	388166
cg17103217	0.06367507	-0.0611472	6.23E-11	37	6	33245721	B3GALT4	NM_003782	1stExon	chr6:332446	S_Shore	NA	NA	TRUE	6:33352687-	6:33244505-	Promoter_As	TRUE	313493
cg09626867	0.06197012	-0.0355915	6.73E-11	37	3	45053351	EXOSC7	NR_023353	Body	NA	NA	NA	NA	TRUE	NA	NA	NA	TRUE	184298
cg06578342	0.05472739	-0.0202282	7.12E-11	37	16	4349215	NA	NA	NA	NA	NA	NA	NA	TRUE	NA	NA	NA	NA	128305
cg01289541	-0.0519838	0.04766022	7.43E-11	37	3	170282058	SLC7A14	NM_020949	5'UTR	NA	NA	NA	NA	TRUE	NA	NA	NA	NA	26926
cg24911837	-0.0742765	0.07458277	8.06E-11	37	7	65227864	CCT6P1	NR_003110	Body	chr7:652293	N_Shore	NA	NA	NA	NA	NA	NA	NA	436957
cg06357748	0.11456672	-0.1176304	8.57E-11	37	12	1025529	RAD52	NM_134424	Body	NA	NA	NA	NA	NA	12:895636-8	12:1025443-	Gene_Associ	NA	124327
cg00867472	0.06502921	-0.0641464	8.70E-11	37	1	156714808	HDGF;HDGF;	NM_001126	Body;Body;Body	chr1:156710	S_Shelf	NA	NA	NA	NA	NA	NA	NA	18280
cg03721978	0.05433248	-0.0476476	8.85E-11	37	6	33245706	B3GALT4	NM_003782	1stExon	chr6:332446	S_Shore	NA	NA	TRUE	6:33352687-	6:33244505-	Promoter_As	TRUE	75196
cg07336872	-0.1126657	0.12254128	9.03E-11	37	6	150393535	NA	NA	NA	chr6:150389	S_Shelf	NA	NA	NA	NA	6:150393377	Unclassified_	TRUE	143314
cg08006309	0.07742829	-0.0917804	9.86E-11	37	16	1587810	IFT140;TMEN	NM_014714;	Body;Body	chr16:15876	Island	NA	NA	NA	16:1527106-	16:1587536-	Unclassified_	NA	155520
cg26126879	0.07509709	-0.0638828	1.01E-10	37	8	103916295	NA	NA	NA	NA	NA	NA	NA	TRUE	NA	NA	NA	NA	455859
cg01292810	-0.1335529	0.15135484	1.02E-10	37	3	184241840	NA	NA	NA	chr3:184243	N_Shore	NA	NA	NA	NA	NA	NA	TRUE	27008
cg16536918	0.06470843	-0.0525185	1.09E-10	37	20	3065403	AVP	NM_000490	TSS200	chr20:30630	S_Shore	NA	NA	NA	NA	NA	NA	NA	304010
cg03185794	-0.0724857	0.05709307	1.18E-10	37	13	101237439	NA	NA	NA	chr13:10124	N_Shelf	NA	NA	NA	NA	13:10123707	Unclassified_	NA	64690
cg03848580	0.08279338	-0.067649	1.19E-10	37	17	76113713	TMC6;TMC6	NM_00112	Body;Body	NA	NA	NA	NA	NA	17:73625246	17:76113116	Unclassified	TRUE	77552
cg23661483	0.09203007	-0.0742422	1.21E-10	37	19	15227219	ILVBL	NM_006844	Body	chr19:15224	S_Shelf	NA	NA	NA	NA	NA	NA	NA	416503
cg08843623	0.09841637	-0.0887876	1.27E-10	37	16	85627648	NA	NA	NA	NA	NA	NA	NA	TRUE	NA	NA	NA	NA	170596
cg08384239	0.06009976	-0.050803	1.27E-10	37	2	160041968	TANC1;TANC	NM_033394;	Body;Body	NA	NA	NA	NA	TRUE	NA	2:160041913	Unclassified_	TRUE	162549
cg08323969	0.06745321	-0.0422378	1.28E-10	37	17	40770823	NA	NA	NA	NA	NA	NA	NA	TRUE	NA	17:40770556	Unclassified_	TRUE	161523
cg07803375	0.07653202	-0.0556612	1.32E-10	37	7	811206	HEATR2	NM_017802	Body	chr7:813627-	N_Shelf	NA	NA	TRUE	7:777720-77	NA	NA	NA	151848
cg22098511	0.03110176	-0.0345797	1.39E-10	37	17	79476521	NA	NA	NA	chr17:79478	N_Shore	NA	RDMR	NA	NA	17:79476269	Promoter_As	NA	391849
cg00711496	-0.1447868	0.1405205	1.43E-10	37	19	50191497	C19orf76;PR	NM_001101	TSS1500;Body;Body;Bo	chr19:50191	Island	NA	NA	NA	NA	NA	NA	NA	15037
cg03127244	0.07917321	-0.0768296	1.48E-10	37	6	33245638	B3GALT4	NM_003782	1stExon	chr6:332446	S_Shore	NA	NA	TRUE	6:33352687-	6:33244505-	Promoter_As	TRUE	63542
cg19377250	0.06722725	-0.0453553	1.58E-10	37	7	100463206	SLC12A9	NM_020246	Body	chr7:100463	N_Shore	NA	CDMR	NA	NA	NA	NA	NA	349673
cg09022230	0.09988697	-0.0902687	1.58E-10	37	7	5457225	TNRC18	NM_001080	Body	chr7:545850	N_Shore	NA	NA	NA	NA	NA	NA	NA	173796
cg04761746	-0.0832354	0.10789253	1.60E-10	37	4	141177756	SCOC	NM_032547	TSS1500	chr4:141173	S_Shelf	NA	NA	NA	NA	NA	NA	TRUE	95027
cg19075787	0.07219177	-0.0366969	1.69E-10	37	2	105372087	NA	NA	NA	NA	NA	NA	NA	NA	2:104738513	NA	NA	NA	345234
cg02597894	0.08037379	-0.0672562	2.02E-10	37	6	31760796	VARS	NM_006295	Body	chr6:317632	N_Shelf	NA	NA	NA	NA	NA	NA	NA	52896
cg17200690	0.07858407	-0.0806387	2.04E-10	37	6	33245619	B3GALT4	NM_003782	1stExon	chr6:332446	S_Shore	NA	NA	TRUE	6:33352687-	6:33244505-	Promoter_As	TRUE	315149
cg13027206	0.05649903	-0.0669124	2.10E-10	37	14	91866325	CCDC88C	NM_001080	Body	NA	NA	NA	DMR	NA	NA	14:91866155	Unclassified_	NA	240844
cg06507987	0.06389174	-0.0682857	2.26E-10	37	1	27686768	MAP3K6	NM_004672	Body	chr1:276870	N_Shore	NA	NA	NA	NA	NA	NA	NA	126967
cg18650367	-0.1016524	0.09844569	2.27E-10	37	10	133909949	NA	NA	NA	NA	NA	NA	DMR	NA	NA	NA	NA	NA	338638
cg13583523	-0.0567286	0.0324186	2.30E-10	37	5	24764190	NA	NA	NA	NA	NA	NA	NA	TRUE	NA	NA	NA	NA	250860
cg09646173	-0.0734968	0.06809889	2.32E-10	37	5	149317669	PDE6A	NM_000440	Body	NA	NA	NA	NA	TRUE	NA	NA	NA	NA	184623
cg08709672	0.06567562	-0.0557973	2.34E-10	37	1	206224334	AVPR1B;AVP	NM_000707;	5'UTR;1stExon	chr1:206223	S_Shore	NA	NA	NA	NA	NA	NA	TRUE	168291
cg19567594	0.07378341	-0.0554543	2.34E-10	37	11	10715175	MRVI1;MRV	NM_001100	1stExon;5'UTR;1stExon	NA	NA	low-CpG:106	NA	TRUE	NA	NA	NA	NA	352747
cg20454518	0.05791901	-0.0472717	2.36E-10	37	12	133135463	FBRSL1	NM_001142	Body	chr12:13313	Island	NA	NA	NA	12:13164508	12:13313439	Unclassified_	NA	366979
cg13557167	0.06479337	-0.0459657	2.53E-10	37	17	4840493	SLC25A11;SL	NM_00116	3'UTR;3'UTR;3'UTR	chr17:48428	N_Shelf	NA	NA	NA	NA	NA	NA	NA	250237
cg05512561	-0.1444719	0.17503453	2.53E-10	37	13	33925656	NA	NA	NA	chr13:33923	S_Shore	NA	NA	NA	NA	NA	NA	TRUE	109375

cg17393635	0.06615922	-0.057821	2.54E-10	37	19	49843565	CD37;CD37	NM_001040(	Body;Body	chr19:498426	Island	NA	NA	TRUE	19:54534295	19:49843436	Unclassified	NA	318337
cg00461022	0.05317478	-0.0680938	2.57E-10	37	5	16618052	FAM134B	NM_001034	TSS1500	chr5:1661650	S_Shore	NA	NA	NA	NA	NA	NA	NA	9749
cg20952257	-0.0936782	0.11454062	2.62E-10	37	5	171074407	NA	NA	NA	NA	NA	NA	NA	TRUE	NA	NA	NA	TRUE	374486
cg11388320	0.09969872	-0.080936	2.95E-10	37	4	81119299	PRDM8;PRDI	NM_020226;	5'UTR;5'UTR	chr4:811190	Island	NA	NA	NA	4:81338101-	NA	NA	NA	214344
cg13787850	-0.1014034	0.11549754	2.96E-10	37	9	102195951	NA	NA	NA	NA	NA	NA	NA	TRUE	NA	9:102195826	Unclassified	TRUE	254994
cg13378649	0.09557518	-0.0538332	2.96E-10	37	1	222927856	NA	NA	NA	NA	NA	NA	NA	TRUE	NA	NA	NA	TRUE	246463
cg02216481	0.06073017	-0.0282395	3.00E-10	37	17	7942137	ALOX15B;AL	NM_001039	TSS1500;TSS1500;TSS1	NA	NA	NA	NA	NA	NA	NA	NA	NA	45254
cg20634227	0.05913989	-0.0372166	3.01E-10	37	22	50921009	ADM2	NM_024866	Body	chr22:509194	S_Shore	NA	NA	TRUE	NA	22:50919485	Unclassified	TRUE	369636
cg00078456	0.07291066	-0.063025	3.13E-10	37	1	1564422	MIB2;MIB2;M	NM_001170	Body;Body;Body;Body;B	chr1:156442	Island	NA	NA	NA	NA	NA	NA	NA	1809
cg00653615	0.03889491	-0.0315765	3.24E-10	37	1	151029327	CDC42SE1;CD	NM_001038	5'UTR;5'UTR	chr1:151031	N_Shelf	NA	NA	NA	NA	1:151028715	Promoter_As	NA	13843
cg08899895	0.09042115	-0.0833377	3.25E-10	37	16	85684853	KIAA0182;KI	NM_001134	Body;Body	chr16:85684	S_Shore	NA	NA	NA	16:84241637	16:85684742	Unclassified	NA	171764
cg20164016	0.05862226	-0.0434977	3.29E-10	37	1	175474471	TNR	NM_003285	5'UTR	NA	NA	NA	NA	NA	1:173740992	NA	NA	NA	362724
cg08305942	-0.1510063	0.15318904	3.43E-10	37	16	79692354	NA	NA	NA	NA	NA	NA	NA	NA	16:78249855	NA	NA	NA	161196
cg05903330	0.06335271	-0.0457774	3.49E-10	37	22	39542136	CBX7	NM_175709	Body	chr22:395410	Island	NA	NA	NA	NA	NA	NA	NA	116356
cg04235768	0.06223388	-0.0509208	3.53E-10	37	4	81118343	PRDM8;PRDI	NM_001099	TSS1500;5'UTR	chr4:811181	Island	NA	NA	NA	4:81336961-	NA	NA	NA	85109
cg01801603	0.06188492	-0.0668734	3.59E-10	37	8	123795433	ZHX2	NM_014943	5'UTR	chr8:123792	S_Shore	NA	CDMR	NA	NA	8:123795401	Promoter_As	NA	36897
cg23235965	0.07796882	-0.0504498	3.68E-10	37	6	30459540	HLA-E	NM_005516	Body	chr6:304573	S_Shore	NA	NA	NA	NA	NA	NA	NA	409607
cg08214927	0.05391722	-0.0408764	3.76E-10	37	14	91765824	CCDC88C	NM_001080	Body	NA	NA	NA	NA	NA	14:90835527	NA	NA	NA	159428
cg12590902	0.08251349	-0.074558	3.86E-10	37	1	44771985	ERI3	NM_024066	Body	NA	NA	NA	NA	NA	NA	1:44771813-	Gene_Associ	NA	234120
cg15095906	0.05636926	-0.0350701	3.94E-10	37	1	36789786	FAM176B	NM_018166	TSS200	chr1:367865	S_Shore	NA	NA	NA	NA	NA	NA	NA	280311
cg23025459	0.09559568	-0.0844054	3.96E-10	37	1	3134420	PRDM16;PRI	NM_022114	Body;Body	NA	NA	NA	NA	NA	1:3123982-3	NA	NA	NA	406028
cg04523868	0.05117195	-0.0219784	4.07E-10	37	11	64563983	MAP4K2	NM_004579	Body	NA	NA	NA	NA	NA	NA	NA	NA	NA	90636
cg24448421	0.06634623	-0.0583897	4.44E-10	37	12	68737061	NA	NA	NA	NA	NA	NA	NA	TRUE	NA	NA	NA	NA	429128
cg04604946	0.04296207	-0.035712	4.46E-10	37	12	7023352	LRRC23;ENO	NM_201650	3'UTR;TSS1500;3'UTR;	chr12:70232	Island	NA	CDMR	NA	12:6893563-	12:7023192-	Promoter_As	NA	92204
cg02676175	0.15120902	-0.1218661	4.67E-10	37	17	7253720	KCTD11;ACA	NM_001002	TSS1500;Body	chr17:72532	Island	NA	NA	NA	17:7194023-	17:7253029-	Promoter_As	NA	54546
cg10239319	0.07074185	-0.0542302	4.79E-10	37	4	6073656	JAKMIP1;JAK	NM_144720	Body;Body	NA	NA	NA	NA	TRUE	NA	NA	NA	NA	195028
cg08003402	-0.0730903	0.09849133	4.99E-10	37	3	15311021	SH3BP5;SH3	NM_001018	Body;Body	NA	NA	NA	NA	TRUE	NA	3:15310982-	Promoter_As	TRUE	155473
cg06490845	0.05142717	-0.0413866	5.19E-10	37	16	88858315	NA	NA	NA	NA	NA	NA	NA	TRUE	16:87385690	16:88857645	Unclassified	NA	126653
cg26337070	0.10010255	-0.0635744	5.40E-10	37	2	85999873	ATOH8	NM_032827	Body	NA	NA	NA	NA	TRUE	2:85852898-	NA	NA	NA	459577
cg01748892	0.06801249	-0.049836	5.44E-10	37	7	27184667	HOXA5	NM_019102	TSS1500	chr7:271826	Island	NA	NA	NA	7:27150952-	NA	NA	NA	35782
cg16846518	-0.0965343	0.11777727	5.53E-10	37	3	128062608	EEFSEC	NM_021937	Body	NA	NA	NA	NA	TRUE	NA	NA	NA	TRUE	309525
cg06753439	0.07337427	-0.0567643	5.77E-10	37	6	33245488	B3GALT4	NM_003782	1stExon	chr6:332446	Island	NA	NA	TRUE	6:33352687-	6:33244505-	Promoter_As	TRUE	131658
cg27106909	0.06517408	-0.0621124	5.95E-10	37	16	30106897	YPEL3;YPEL3	NM_031477	1stExon;5'UTR;5'UTR	chr16:30107	N_Shore	NA	NA	TRUE	16:30014183	16:30106816	Promoter_As	NA	472978
cg24124954	0.06580839	-0.0586379	6.01E-10	37	6	31508106	BAT1;BAT1	NM_080598	Body;Body	chr6:315096	N_Shore	NA	RDMR	NA	NA	NA	NA	NA	424083
cg00138407	0.06086783	-0.0377876	6.07E-10	37	3	47386505	KLHL18	NM_025010	3'UTR	NA	NA	NA	NA	NA	NA	NA	NA	NA	3124
cg18563886	-0.0669786	0.03932684	6.13E-10	37	10	135203102	PAOX;PAOX;	NM_207128	Body;Body;3'UTR	chr10:13520	Island	NA	NA	NA	10:13505305	10:13520226	Promoter_As	NA	337268
cg22294740	0.11608971	-0.1060699	6.22E-10	37	19	2294961	LINGO3	NM_001101	5'UTR	chr19:22948	Island	NA	NA	NA	19:2245859-	NA	NA	TRUE	394727
cg11360522	0.04046835	-0.0314505	6.28E-10	37	13	113379828	ATP11A;ATP	NM_015205	Body;Body	chr13:11338	N_Shelf	NA	NA	TRUE	NA	13:11337957	Unclassified	TRUE	213891
cg11419304	-0.0528252	0.0417601	6.32E-10	37	2	69248344	ANTXR1;AN	NM_053034	Body;Body;Body	NA	NA	NA	NA	TRUE	NA	NA	NA	NA	214916
cg26860970	0.05450371	-0.0300656	6.41E-10	37	6	30881658	VARS2;VARS	NM_001167	TSS1500;TSS1500;TSS1	chr6:308815	Island	NA	NA	NA	6:30989539-	6:30881429-	Promoter_As	NA	468676
cg24247537	0.13287357	-0.1149803	6.51E-10	37	11	457278	PTDSS2	NM_030783	Body	chr11:45908	N_Shore	NA	NA	NA	11:447256-4	11:456465-4	Unclassified	NA	426002
cg06373870	0.08095944	-0.0702853	7.04E-10	37	4	81117853	PRDM8;PRDI	NM_001099	TSS1500;5'UTR	chr4:811181	N_Shore	NA	RDMR	TRUE	4:81336689-	4:81117576-	Unclassified	NA	124647
cg03796381	-0.0504546	0.04891006	7.10E-10	37	15	36650033	NA	NA	NA	NA	NA	NA	NA	TRUE	NA	NA	NA	NA	76648
cg26161820	0.06288561	-0.036605	7.33E-10	37	17	37792777	STARD3;PPP	NM_006804	TSS1500;3'UTR;3'UTR;	chr17:37793	N_Shore	NA	NA	NA	NA	17:37792752	Promoter_As	NA	456520
cg05677712	0.05565654	-0.0387345	7.60E-10	37	3	195586796	NA	NA	NA	NA	NA	NA	NA	TRUE	3:197071193	3:195585913	Unclassified	NA	112351
cg23663547	0.04323491	-0.0359425	7.61E-10	37	17	38710320	CCR7	NM_001838	3'UTR	NA	NA	NA	NA	NA	NA	NA	NA	NA	416535
cg26311932	0.04150237	-0.038546	7.73E-10	37	6	31549563	LTB;LTB	NM_002341	Body;Body	chr6:315484	S_Shore	NA	NA	NA	NA	6:31547692-	Promoter_As	NA	459115
cg05452645	0.08853253	-0.0757539	7.91E-10	37	4	81117647	PRDM8;PRDI	NM_001099	TSS1500;5'UTR	chr4:811181	N_Shore	NA	CDMR	TRUE	NA	4:81117576-	Unclassified	NA	108260
cg24844545	0.04061719	-0.0367783	8.04E-10	37	1	11908347	NPPA	NM_006172	TSS1500	NA	NA	NA	NA	TRUE	NA	NA	NA	NA	435746
cg11701868	0.13723796	-0.1467899	8.24E-10	37	19	4912006	UHRF1;UHR	NM_013282	Body;Body	chr19:49092	S_Shore	NA	RDMR	TRUE	19:4862547-	19:4911827-	Promoter_As	NA	219334
cg13518625	-0.0964697	0.09052739	8.31E-10	37	8	29522838	NA	NA	NA	NA	NA	NA	NA	TRUE	NA	8:29522663-	Unclassified	TRUE	249424
cg08098420	-0.0554733	0.03707919	8.67E-10	37	16	66164601	NA	NA	NA	NA	NA	NA	NA	TRUE	NA	NA	NA	NA	157150
cg17397159	0.05273706	-0.0397499	9.19E-10	37	4	720144	PCGF3	NM_006315	5'UTR	chr4:716281	S_Shelf	NA	NA	TRUE	4:710112-71	NA	NA	TRUE	318387
cg19882268	0.02963004	-0.0219997	9.46E-10	37	6	33245779	B3GALT4	NM_003782	1stExon	chr6:332446	S_Shore	NA	NA	TRUE	6:33352687-	6:33244505-	Promoter_As	NA	358254
cg11093760	0.06682421	-0.0510033	9.58E-10	37	15	65503807	CILP;CILP	NM_003613	5'UTR;1stExon	NA	NA	NA	NA	TRUE	NA	15:65503680	Unclassified	NA	209433
cg06457011	0.12470172	-0.1355739	9.81E-10	37	20	39767490	PLCG1;PLCG	NM_002660	Body;Body	chr20:39765	S_Shore	NA	RDMR	NA	NA	NA	NA	NA	126055
cg16736826	-0.0734139	0.07177178	9.85E-10	37	1	41951512	EDN2	NM_001956	TSS1500	NA	NA	NA	NA	TRUE	NA	NA	NA	TRUE	307826
cg02988947	0.05147799	-0.0581462	9.92E-10	37	17	61778813	LIMD2	NM_030576	TSS1500	chr17:61776	S_Shore	NA	NA	NA	NA	NA	NA	NA	60891
cg07338715	0.04996369	-0.0343941	1.01E-09	37	2	109649281	NA	NA	NA	chr2:109647	S_Shore	NA	RDMR	NA	NA	NA	NA	NA	143354
cg01542019	0.07589654	-0.0711544	1.05E-09	37	19	14673053	TECR	NM_138501	Body	chr19:14676	N_Shelf	NA	NA	TRUE	NA	NA	NA	TRUE	31904
cg18791929	0.0850778	-0.0372719	1.08E-09	37	3	108205828	MYH15	NM_014981	Body	NA	NA	NA	NA	TRUE	NA	NA	NA	NA	340884
cg02721693	-0.1025369	0.10374688	1.12E-09	37	1	182922705	C1orf14	NM_030933	TSS200	chr1:182921	S_Shore	NA	NA	NA	NA	NA	NA	NA	55428
cg03040622	0.04884692	-0.0301801	1.13E-09	37	7	5536937	MIR589;FBX	NR_030318;	TSS1500;Body	chr7:553995	N_Shelf	NA	NA	NA	7:5503439-5	7:5536936-5	Unclassified	NA	61904
cg08899667	0.10571564	-0.0852412	1.16E-09	37	6	31761055	VARS	NM_006295	Body	chr6:317632	N_Shelf	NA	NA	NA	NA	NA	NA	NA	171762
cg18091385	0.07064177	-0.0322278	1.18E-09	37	14	99502466	NA	NA	NA	NA	NA	NA	NA	TRUE	NA	NA	NA	TRUE	329309
cg11381564	0.06950378	-0.0695768	1.18E-09	37	6	32808619	PSMB8;PSM	NM_004159	3'UTR;3'UTR	chr6:328062	S_Shore	NA	NA	NA	NA	NA	NA	NA	214222
cg08115371	0.10399526	-0.0654163	1.18E-09	37	11	79115706	ODZ4	NM_001098	5'UTR	NA	NA	NA	NA	TRUE	NA	NA	NA	NA	157491

cg16641055	0.06012661	-0.055457	1.19E-09	37	16	88846292	FAM38A	NM_001142	Body	chr16:88844	S_Shore	NA	NA	TRUE	16:87373715	NA	NA	NA	305973
cg02452500	0.05062005	-0.0381071	1.27E-09	37	11	13161927	NA	NA	NA	NA	NA	NA	NA	TRUE	NA	11:13161852	Unclassified_	NA	49921
cg01894038	0.0959704	-0.0563284	1.29E-09	37	11	77741873	NA	NA	NA	NA	NA	NA	NA	TRUE	NA	NA	NA	TRUE	38806
cg02619205	0.04354103	-0.0171192	1.30E-09	37	9	117157157	AKNA	NM_030767	TSS1500	chr9:117160	N_Shelf	NA	NA	NA	NA	NA	NA	NA	53341
cg06997114	0.05652462	-0.0300543	1.30E-09	37	18	21851455	OSBPL1A;OS	NM_080597;	Body;5'UTR	chr18:21851	Island	NA	NA	NA	18:20105359	NA	NA	NA	136654
cg14997707	-0.0711956	0.05866743	1.31E-09	37	7	93853120	NA	NA	NA	NA	NA	NA	NA	TRUE	NA	7:93852772-	Unclassified_	NA	278636
cg04247152	0.16103474	-0.1125961	1.34E-09	37	16	88590325	ZFPM1	NM_153813	Body	chr16:88590	Island	NA	NA	NA	16:87117783	NA	NA	NA	85335
cg18877271	0.09671561	-0.071109	1.35E-09	37	11	1952638	TNNT3;TNNT	NM_001042	Body;Body;Body;Body	chr11:19503	S_Shore	NA	NA	NA	11:1909189-	NA	NA	NA	342316
cg19358373	0.04979743	-0.0318218	1.42E-09	37	12	133414571	NA	NA	NA	chr12:13341	S_Shore	NA	NA	NA	NA	NA	NA	NA	349347
cg23090529	-0.0892371	0.06482845	1.42E-09	37	1	51442133	NA	NA	NA	chr1:514433	N_Shore	NA	RDMR	NA	NA	NA	NA	NA	407226
cg24046155	0.06012701	-0.0460398	1.43E-09	37	19	2294369	LINGO3	NM_001101	5'UTR	chr19:22948	N_Shore	NA	DMR	NA	19:2245323-	NA	NA	TRUE	422764
cg18702971	0.0578824	-0.0346072	1.48E-09	37	22	29920546	THOC5;THOC	NM_001002	Body;Body;Body;Body	NA	NA	NA	NA	NA	NA	22:29920492	Gene_Associ	NA	339381
cg09415366	-0.0637739	0.06011528	1.52E-09	37	10	134385407	INPP5A	NM_005539	Body	NA	NA	NA	NA	TRUE	NA	10:13438523	Promoter_As	TRUE	180609
cg07377178	0.05188967	0.00291586	1.54E-09	37	6	3025064	NA	NA	NA	NA	NA	NA	NA	TRUE	NA	NA	NA	TRUE	144046
cg26038649	0.0502514	-0.0436061	1.62E-09	37	1	27683501	MAP3K6	NM_004672	Body	chr1:276832	Island	NA	NA	NA	1:27555571-	1:27683199-	Promoter_As	NA	454404
cg25021247	0.0644312	-0.0463637	1.83E-09	37	3	49460162	AMT;AMT;AI	NR_028435;	TSS200;TSS200;TSS200	NA	NA	NA	NA	TRUE	NA	NA	NA	NA	438717
cg23507953	0.05118097	-0.0407976	1.83E-09	37	17	46638127	HOXB3	NM_002146	5'UTR	chr17:46641	N_Shelf	NA	NA	TRUE	NA	17:46638064	Unclassified_	NA	414032
cg03710029	0.04862849	-0.0578517	1.84E-09	37	17	79265601	SLC38A10;SL	NM_001037	Body;Body	chr17:79268	N_Shelf	NA	NA	NA	17:76879988	17:79265286	Unclassified_	TRUE	74932
cg22907189	0.04726384	-0.0304176	1.85E-09	37	5	172430563	ATP6V0E1	NM_003945	Body	NA	NA	NA	NA	TRUE	NA	NA	NA	NA	404092
cg24807889	-0.0716148	0.06770569	1.99E-09	37	1	1135955	NA	NA	NA	chr1:113636	N_Shore	NA	NA	NA	NA	1:1135946-1	Unclassified	NA	435187
cg06744585	0.09628312	-0.0948994	2.11E-09	37	19	13135808	NFIX	NM_002501	Body	chr19:13135	Island	low-CpG:129	NA	NA	19:12996280	NA	NA	NA	131467
cg15052747	0.03989975	-0.0242502	2.23E-09	37	9	138942591	NACC2	NM_144653	5'UTR	chr9:138941	Island	NA	NA	NA	9:138081292	NA	NA	NA	279546
cg13790576	-0.0704896	0.05999753	2.23E-09	37	9	139640661	LCN6	NM_198946	Body	chr9:139640	Island	NA	NA	NA	9:138760319	NA	NA	TRUE	255056
cg09446583	0.04537937	-0.0327836	2.23E-09	37	2	43398271	NA	NA	NA	chr2:433980	Island	NA	NA	NA	NA	2:43398234-	Unclassified	NA	181175
cg25921609	0.14418809	-0.1315192	2.27E-09	37	17	8379225	MYH10	NM_005964	Body	chr17:83801	N_Shore	NA	NA	NA	17:8319758-	NA	NA	NA	452437
cg18828619	0.05463468	-0.0331459	2.27E-09	37	14	56615077	PELI2	NM_021255	Body	NA	NA	NA	NA	TRUE	NA	NA	NA	NA	341522
cg11993160	-0.0856717	0.10457698	2.28E-09	37	18	12404041	NA	NA	NA	chr18:12407	N_Shelf	NA	NA	NA	NA	NA	NA	TRUE	224500
cg17400113	0.05522684	-0.0361756	2.48E-09	37	15	93617146	RGMA;RGM/	NM_001166	1stExon;TSS1500;Body	chr15:93615	Island	NA	NA	NA	15:91416627	15:93616879	Unclassified_	NA	318434
cg06776173	0.05309359	-0.0313895	2.50E-09	37	1	46932780	NA	NA	NA	chr1:469321	Island	NA	NA	TRUE	1:46704650-	1:46931908-	Unclassified_	TRUE	132148
cg24217948	-0.0553408	0.05662125	2.50E-09	37	18	42261980	SETBP1;SETE	NM_001130	5'UTR;5'UTR	chr18:42258	S_Shore	NA	RDMR	TRUE	NA	NA	NA	NA	425640
cg07925670	0.11168457	-0.0855865	2.58E-09	37	17	7253489	ACAP1	NM_014716	Body	chr17:72532	Island	NA	NA	NA	17:7194023-	17:7253029-	Promoter_As	NA	154106
cg13430552	0.05503353	-0.0534975	2.71E-09	37	2	25427652	NA	NA	NA	chr2:254271	S_Shore	NA	NA	NA	NA	2:25427162-	Unclassified_	NA	247495
cg05915362	-0.0662653	0.07331545	2.72E-09	37	6	159274480	OSTCL	NR_028496	Body	NA	NA	NA	NA	TRUE	NA	6:159274295	Promoter_As	TRUE	116567
cg08750510	-0.0885488	0.06223902	2.82E-09	37	5	32537617	NA	NA	NA	NA	NA	NA	NA	TRUE	NA	NA	NA	NA	169007
cg01368217	0.05950369	-0.037131	2.83E-09	37	10	127836603	ADAM12;AD/	NM_003474;	Body;Body	NA	NA	NA	NA	TRUE	NA	NA	NA	NA	28439
cg19880597	0.0727124	-0.0485752	2.83E-09	37	5	136965606	KLHL3	NM_017415	Body	NA	NA	NA	NA	TRUE	NA	5:136964818	Unclassified	TRUE	358223
cg24556382	-0.1022536	0.10903367	2.86E-09	37	4	174173455	GALNT7	NM_017423	Body	NA	NA	NA	NA	TRUE	NA	NA	NA	TRUE	430977
cg03131366	0.05921835	-0.0350475	2.90E-09	37	12	2041707	NA	NA	NA	NA	NA	NA	NA	NA	12:1911668-	NA	NA	NA	63637
cg23732024	-0.0864421	0.06946106	2.94E-09	37	8	74903801	LY96	NM_015364	Body	NA	NA	NA	NA	TRUE	NA	NA	NA	NA	417774
cg08818610	0.10921114	-0.0978156	2.98E-09	37	6	24910720	FAM65B	NM_014722	5'UTR	chr6:249106	Island	NA	DMR	NA	6:25018606-	NA	NA	NA	170119
cg00984696	-0.0531038	0.03971466	2.98E-09	37	3	124128381	KALRN;KALR	NM_001024	Body;Body	NA	NA	NA	NA	TRUE	NA	NA	NA	NA	20709
cg22539182	0.07203306	-0.0508217	3.04E-09	37	10	850393	NA	NA	NA	chr10:85110	N_Shore	NA	NA	TRUE	NA	10:850372-8	Unclassified	NA	398228
cg06812840	0.05117399	-0.0427015	3.18E-09	37	17	7492524	SOX15	NM_006942	1stExon	chr17:74923	Island	NA	NA	NA	17:7432995-	17:7491270-	Unclassified	NA	132905
cg07677157	-0.1504338	0.14515999	3.19E-09	37	12	66050928	NA	NA	NA	NA	NA	NA	NA	TRUE	NA	NA	NA	TRUE	149585
cg02116864	0.06899252	-0.0663761	3.19E-09	37	10	134222453	PWWP2B;PV	NM_138499;	3'UTR;Body	chr10:13421	S_Shelf	NA	NA	NA	10:13407240	NA	NA	NA	43449
cg16014606	0.08355164	-0.0746775	3.26E-09	37	11	10715221	MRVI1;MRVI	NM_001100	1stExon;5'UTR;1stExon	NA	NA	low-CpG:106	NA	TRUE	NA	NA	NA	NA	295011
cg03165356	-0.1040054	0.13240956	3.30E-09	37	2	216882347	NA	NA	NA	chr2:216877	S_Shelf	NA	NA	NA	NA	2:216882322	Unclassified_	TRUE	64300
cg21535931	-0.1132368	0.12548705	3.33E-09	37	8	145029603	PLEC1;PLEC1	NM_000445;	Body;Body	chr8:145027	S_Shore	NA	NA	TRUE	NA	NA	NA	NA	383457
cg13553455	-0.0586393	0.05135254	3.35E-09	37	10	105846002	COL17A1	NM_000494	TSS1500	NA	NA	NA	NA	NA	NA	NA	NA	NA	250145
cg17344906	0.08840699	-0.0919413	3.48E-09	37	19	13202507	NFIX	NM_002501	3'UTR	chr19:13198	S_Shelf	NA	NA	NA	NA	19:13202502	Unclassified_	NA	317563
cg04115307	-0.1252777	0.11375227	3.54E-09	37	14	101539612	NA	NA	NA	NA	NA	NA	NA	TRUE	14:10060911	NA	NA	NA	82665
cg01791634	0.06719586	-0.082857	3.57E-09	37	17	76130139	TMC8	NM_152468	Body	chr17:76127	S_Shore	NA	NA	NA	17:73641728	17:76129950	Promoter_As	NA	36675
cg27646484	0.06318651	-0.0475134	3.61E-09	37	16	31210736	NA	NA	NA	chr16:31213	N_Shelf	NA	NA	NA	NA	NA	NA	NA	482082
cg04219321	0.03540909	-0.0240342	3.77E-09	37	17	53341659	HLF	NM_002126	TSS1500	chr17:53342	N_Shore	NA	NA	NA	17:50696658	17:53341444	Unclassified_	NA	84757
cg27164770	-0.1183044	0.10353926	3.78E-09	37	13	94891445	GPC6	NM_005708	Body	NA	NA	NA	NA	TRUE	NA	NA	NA	NA	473950
cg14548802	0.11914115	-0.121633	3.93E-09	37	9	137675380	COL5A1	NM_000093	Body	NA	NA	NA	NA	NA	9:136814982	NA	NA	NA	270731
cg16300030	0.0385706	-0.0338626	4.07E-09	37	6	32908980	HLA-DMB	NM_002118	TSS200	NA	NA	NA	NA	TRUE	NA	6:32908496-	Unclassified	TRUE	299736
cg22670759	0.05701029	-0.0354436	4.09E-09	37	3	50487955	CACNA2D2;C	NM_001005	Body;Body	NA	NA	NA	NA	NA	3:50462908-	3:50487465-	Unclassified_	NA	400108
cg13707760	0.06002815	-0.0453274	4.14E-09	37	9	95986383	WNK2	NM_006648	Body	NA	NA	NA	NA	TRUE	NA	NA	NA	TRUE	253438
cg07807757	-0.1413563	0.17301198	4.14E-09	37	7	50894570	NA	NA	NA	NA	NA	NA	NA	TRUE	NA	NA	NA	TRUE	151952
cg17917325	0.04703427	-0.0321066	4.30E-09	37	7	56142347	SUMF2;SUM	NM_001042	Body;Body;Body;Body;B	NA	NA	NA	NA	NA	7:56109650-	7:56141833-	Gene_Associ	TRUE	326396
cg12810800	-0.1287553	0.14763551	4.34E-09	37	2	159788345	NA	NA	NA	NA	NA	NA	NA	TRUE	NA	NA	NA	NA	237631
cg17640485	0.07834648	-0.0573388	4.44E-09	37	3	195620147	TNK2;TNK2	NM_001010	Body;5'UTR	chr3:195622	N_Shelf	NA	NA	TRUE	NA	NA	NA	TRUE	322158
cg20603222	0.05211114	-0.0353998	4.68E-09	37	7	1096387	C7orf50;C7or	NM_001134	Body;Body;TSS1500;Bo	chr7:109740	N_Shore	NA	NA	NA	7:1062770-1	NA	NA	TRUE	369209
cg09852607	0.04649739	-0.0322319	4.69E-09	37	3	128205200	GATA2;GATA	NM_001145	Body;Body;Body	chr3:128205	N_Shore	NA	RDMR	TRUE	3:129687779	NA	NA	NA	188108
cg23482898	0.05732939	-0.0481475	4.72E-09	37	3	12858887	CAND2;CAND	NM_001162	Body;Body	chr3:128566	S_Shelf	NA	NA	NA	3:12833887-	NA	NA	NA	413610
cg24257309	0.05556729	-0.0445311	4.87E-09	37	20	3065423	AVP	NM_000490	TSS200	chr20:30630	S_Shore	NA	NA	NA	NA	NA	NA	NA	426154

cg14172849	0.10051824	-0.0857924	5.13E-09	37	14	104171259	XRCC3;XRCC	NM_001100	Body;Body;Body	NA	NA	NA	NA	NA	14:10324099	NA	NA	NA	262966
cg12076102	0.05681506	-0.0411709	5.21E-09	37	17	62084322	ICAM2;ICAM	NM_001099	5'UTR;5'UTR;5'UTR;5'U	NA	NA	low-CpG:594	NA	NA	NA	NA	NA	NA	225917
cg03957124	-0.0828969	0.06629897	5.43E-09	37	6	37016869	NA	NA	NA	chr6:370126	S_Shelf	NA	NA	NA	NA	6:37016466-	Unclassified	TRUE	79573
cg23786580	0.04971349	-0.0365113	5.47E-09	37	7	43204506	HECW1	NM_015052	5'UTR	NA	NA	NA	NA	TRUE	NA	7:43204379-	Unclassified	TRUE	418444
cg26361757	0.06026312	-0.0562464	5.53E-09	37	1	6706713	DNAJC11	NM_018198	Body	NA	NA	NA	NA	NA	NA	1:6706534-6	Gene_Associ	TRUE	460066
cg26460530	0.06919797	-0.0738581	5.71E-09	37	22	39919067	NA	NA	NA	chr22:39915	S_Shore	NA	RDMR	NA	NA	22:39918755	Promoter_As	TRUE	461690
cg11405655	-0.045993	0.04647479	5.73E-09	37	1	999599	NA	NA	NA	chr1:999678-	N_Shore	NA	NA	NA	1:989344-98	1:998374-10	NonGene_As	NA	214644
cg13257654	0.05597175	-0.0429682	5.80E-09	37	8	42946331	NA	NA	NA	chr8:429485	N_Shelf	NA	NA	NA	NA	NA	NA	NA	244418
cg08462055	0.07828803	-0.0793558	5.91E-09	37	15	64944023	ZNF609	NM_015042	Body	NA	NA	NA	NA	TRUE	NA	15:64943717	Unclassified	NA	163859
cg10471113	0.09811383	-0.1240005	6.07E-09	37	16	1587842	IFT140;TMEI	NM_014714;	Body;Body	chr16:15876	Island	NA	NA	NA	16:1527106-	16:1587536-	Unclassified	NA	198896
cg11699257	0.06201257	-0.0243415	6.08E-09	37	8	28634455	INTS9;INTS9	NM_018250;	Body;Body;Body	NA	NA	NA	NA	TRUE	NA	8:28634408-	Unclassified	NA	219285
cg00257455	0.05022148	-0.0472203	6.33E-09	37	1	65530538	NA	NA	NA	chr1:655321	N_Shore	NA	NA	NA	NA	NA	NA	NA	5520
cg03746015	-0.1396052	0.14795275	6.35E-09	37	16	11141000	CLEC16A	NM_015226	Body	NA	NA	NA	NA	TRUE	NA	NA	NA	NA	75694
cg01899318	-0.087252	0.07797957	6.36E-09	37	16	71523395	ZNF19	NM_006961	TSS200	NA	NA	NA	NA	NA	16:70080707	NA	NA	NA	38899
cg01751802	0.0678551	-0.0673118	6.59E-09	37	19	11309639	KANK2	NM_001136	TSS1500	chr19:11307	S_Shore	NA	NA	NA	NA	NA	NA	NA	35846
cg12559228	-0.09488	0.09057805	6.59E-09	37	19	50191882	C19orf76	NM_001101	TSS200	chr19:50192	N_Shore	NA	NA	TRUE	NA	NA	NA	NA	233594
cg09152259	-0.0533324	0.075097	6.62E-09	37	2	128156114	NA	NA	NA	chr2:128158	N_Shelf	NA	NA	NA	NA	2:128155907	Unclassified	NA	175977
cg26077811	-0.1124513	0.08902712	6.94E-09	37	11	119232263	USP2;USP2	NM_004205;	Body;Body	chr11:11923	N_Shelf	NA	NA	NA	NA	11:11923223	Unclassified	TRUE	455002
cg05814312	-0.0983765	0.11816537	6.99E-09	37	10	29760687	SVIL;SVIL	NM_021738;	Body;Body	NA	NA	NA	NA	TRUE	NA	NA	NA	TRUE	114704
cg14507310	-0.060973	0.05496377	7.33E-09	37	9	121285184	NA	NA	NA	NA	NA	NA	NA	TRUE	NA	NA	NA	NA	269799
cg06724693	0.05060744	-0.0565421	7.46E-09	37	1	3010896	PRDM16;PRI	NM_022114;	Body;Body	NA	NA	NA	NA	NA	1:3000575-3	NA	NA	NA	131108
cg01157559	0.10293745	-0.0844211	7.47E-09	37	16	29166162	NA	NA	NA	chr16:29169	N_Shelf	NA	NA	TRUE	16:29073495	NA	NA	NA	24385
cg21325715	-0.0744853	0.07343489	7.48E-09	37	17	80297128	NA	NA	NA	NA	NA	NA	NA	TRUE	17:77890417	NA	NA	TRUE	380481
cg26299044	0.1456224	-0.0889675	7.48E-09	37	17	39021588	KRT12	NM_000223	Body	chr17:39019	S_Shore	NA	NA	NA	NA	NA	NA	NA	458880
cg09735822	0.05866393	-0.0490152	7.50E-09	37	12	133015059	NA	NA	NA	chr12:13301	N_Shore	NA	NA	NA	12:13152495	NA	NA	NA	186097
cg16348158	0.06274617	-0.0484266	7.55E-09	37	1	3036916	PRDM16;PRI	NM_022114;	Body;Body	chr1:303806	N_Shore	NA	NA	NA	1:3026721-3	NA	NA	NA	300606
cg00581154	0.06408306	-0.0462851	7.98E-09	37	1	3123695	PRDM16;PRI	NM_022114;	Body;Body	NA	NA	NA	NA	NA	1:3113154-3	NA	NA	NA	12384
cg18740175	0.06314474	-0.0768545	8.16E-09	37	20	50110833	NFATC2;NFA	NM_001136	Body;Body;Body	chr20:50108	S_Shore	NA	NA	NA	NA	20:50110492	Promoter_As	NA	339982
cg15261712	0.06990203	-0.0688923	8.31E-09	37	7	92238248	CDK6;CDK6	NM_001259;	3'UTR;3'UTR	NA	NA	NA	CDMR	NA	NA	NA	NA	NA	282910
cg24049493	0.08982981	-0.0876751	8.33E-09	37	1	42385941	HIVEP3;HIVE	NM_024503;	TSS1500;TSS1500	chr1:423836	S_Shore	NA	RDMR	NA	NA	1:42385318-	Promoter_As	NA	422820
cg06791426	0.04769798	-0.0486772	8.48E-09	37	12	133135361	FBRSL1	NM_001142	Body	chr12:13313	Island	NA	NA	NA	12:13164508	12:13313439	Unclassified	NA	132457
cg00686915	0.07178301	-0.0417823	8.87E-09	37	7	99076604	ZNF789;ZNF	NM_001013	Body;Body	NA	NA	NA	NA	NA	NA	7:99076509-	Promoter_As	NA	14489
cg09098522	0.05742543	-0.0193822	8.92E-09	37	10	31435673	NA	NA	NA	NA	NA	NA	NA	TRUE	10:31475651	NA	NA	NA	175031
cg06753787	0.0517263	-0.0263477	9.25E-09	37	2	220074208	ZFAND2B	NM_138802	3'UTR	chr2:220071	S_Shelf	NA	NA	NA	NA	NA	NA	NA	131663
cg14873022	0.07704183	-0.0359758	9.30E-09	37	4	146841701	ZNF827	NM_178835	Body	NA	NA	NA	NA	NA	NA	NA	NA	TRUE	276612
cg09349343	0.0508422	-0.0469572	9.36E-09	37	6	33245717	B3GALT4	NM_003782	1stExon	chr6:332446	S_Shore	NA	NA	TRUE	6:33352687-	6:33244505-	Promoter_As	TRUE	179444
cg19409579	0.11730148	-0.0996669	9.44E-09	37	4	81118500	PRDM8;PRDI	NM_020226;	5'UTR;TSS200	chr4:811181	Island	NA	NA	NA	4:81336961-	4:81118358-	Unclassified	NA	350194
cg10453071	0.04862244	-0.0259726	9.48E-09	37	16	57513414	DOK4	NM_018110	Body	NA	NA	NA	NA	TRUE	16:56070737	NA	NA	NA	198606
cg21946195	0.04396948	-0.0126419	9.48E-09	37	2	86012225	ATOH8	NM_032827	Body	NA	NA	NA	NA	TRUE	NA	NA	NA	TRUE	389751
cg05945608	0.05226537	-0.0361488	9.56E-09	37	6	42739639	NA	NA	NA	NA	NA	NA	NA	TRUE	NA	6:42739603-	Promoter_As	NA	117213
cg13244312	0.03317613	-0.0284134	9.61E-09	37	14	71109707	TTC9	NM_015351	Body	chr14:71108	S_Shore	NA	RDMR	NA	NA	NA	NA	NA	244208
cg19679112	0.04094224	-0.0171776	9.74E-09	37	17	30333737	NA	NA	NA	chr17:30333	N_Shore	NA	NA	NA	NA	17:30333558	Promoter_As	NA	354596
cg15393490	0.05603455	-0.0665845	9.79E-09	37	1	207996459	NA	NA	NA	NA	NA	NA	CDMR	TRUE	NA	1:207995893	Promoter_As	TRUE	284769
cg00548098	-0.0864556	0.10517005	9.87E-09	37	16	11295667	NA	NA	NA	NA	NA	NA	NA	TRUE	NA	NA	NA	TRUE	11632
cg23950233	0.08955365	-0.0771024	9.94E-09	37	6	33245739	B3GALT4	NM_003782	1stExon	chr6:332446	S_Shore	NA	NA	TRUE	6:33352687-	6:33244505-	Promoter_As	TRUE	421004
cg01671681	-0.0522442	0.06053301	1.00E-08	37	3	155421735	PLCH1	NM_014996	5'UTR	NA	NA	NA	NA	TRUE	NA	NA	NA	NA	34410
cg02222982	0.06183969	-0.0612594	1.00E-08	37	11	67207599	CORO1B;COI	NM_001018	Body;Body	chr11:67203	S_Shelf	NA	NA	NA	11:66964001	11:67207594	Unclassified	NA	45376
cg18561976	-0.0905596	0.09411298	1.03E-08	37	2	204801508	ICOS;ICOS	NM_012092;	5'UTR;1stExon	NA	NA	NA	NA	NA	NA	2:204801321	Unclassified	NA	337238
cg18534312	0.02854554	-0.0137428	1.03E-08	37	8	140642584	KCNK9	NM_016601	Body	NA	NA	NA	NA	NA	8:140711730	8:140642475	Unclassified	NA	336808
cg10825530	0.09625531	-0.0629191	1.07E-08	37	11	16023708	SOX6;SOX6;S	NM_033326;	Body;Body;Body;Body	NA	NA	NA	NA	TRUE	NA	NA	NA	TRUE	205041
cg09931783	0.09005692	-0.0790246	1.08E-08	37	12	123469669	PITPNM2	NM_020845	3'UTR	chr12:12347	N_Shore	NA	NA	NA	12:12203519	12:12346966	Unclassified	NA	189513
cg17164345	-0.0542719	0.04229896	1.09E-08	37	11	77271314	NA	NA	NA	NA	NA	NA	NA	TRUE	NA	NA	NA	NA	314516
cg04554131	0.05342279	-0.0418714	1.09E-08	37	1	25291540	RUNX3	NM_001031	TSS200	NA	NA	NA	NA	NA	1:25163972-	1:25291322-	Unclassified	NA	91201
cg04681525	0.0374445	-0.0107345	1.09E-08	37	9	132145577	NA	NA	NA	chr9:132145	Island	NA	NA	TRUE	NA	9:132145334	Promoter_As	NA	93564
cg18524934	0.0441409	-0.0369781	1.10E-08	37	2	97164073	NEURL3	NR_026875	Body	chr2:971662	N_Shelf	NA	NA	NA	NA	NA	NA	NA	336645
cg07443710	-0.0959236	0.10605128	1.11E-08	37	10	71206848	NA	NA	NA	NA	NA	NA	NA	TRUE	NA	NA	NA	NA	145190
cg17772649	0.05617992	-0.0307018	1.12E-08	37	6	31691426	C6orf25;C6or	NM_138274;	Body;Body;Body;Body;E	chr6:316914	Island	NA	NA	NA	6:31799014-	NA	NA	NA	324039
cg24927174	0.06448973	-0.0540609	1.14E-08	37	1	154942566	SHC1;SHC1;S	NM_003029;	Body;1stExon;Body;1st	chr1:154946	N_Shelf	NA	NA	NA	NA	NA	NA	NA	437192
cg16348385	0.03473785	-0.0332803	1.15E-08	37	16	30106822	YPEL3;YPEL3	NM_031477;	1stExon;5'UTR;5'UTR	chr16:30107	N_Shore	NA	NA	TRUE	16:30014183	16:30106816	Promoter_As	NA	300612
cg11186344	-0.139281	0.12616358	1.17E-08	37	1	19253694	IFFO2	NM_001136	Body	NA	NA	NA	NA	TRUE	NA	1:19252744-	Promoter_As	TRUE	210894
cg19483007	-0.0650391	0.05011874	1.18E-08	37	3	149327651	WWTR1;WW	NM_015472;	Body;Body;Body	NA	NA	NA	NA	TRUE	NA	3:149327380	Unclassified	NA	351364
cg17178761	0.12477317	-0.102671	1.19E-08	37	17	55682851	MSI2;MSI2	NM_138962;	Body;Body	NA	NA	NA	NA	TRUE	NA	17:55682560	Unclassified	NA	314795
cg06897661	0.11036339	-0.1146066	1.19E-08	37	16	50322074	ADCY7;ADCY	NM_001114;	5'UTR;1stExon	NA	NA	NA	NA	NA	NA	NA	NA	NA	134741
cg03744763	0.07240809	-0.0600715	1.21E-08	37	7	27184737	HOXA5	NM_019102	TSS1500	chr7:271826	Island	NA	NA	NA	7:27150952-	NA	NA	NA	75671
cg00328227	0.04022488	-0.0397875	1.21E-08	37	1	109204325	C1orf59;C1or	NM_144584;	TSS200;TSS1500	chr1:109203	Island	NA	NA	NA	1:109005133	1:109203476	Promoter_As	TRUE	6912
cg07599786	0.05296249	-0.0520846	1.22E-08	37	1	201618654	NAV1	NM_020443	Body	chr1:201617	Island	NA	NA	NA	1:199883665	NA	NA	NA	148238
cg15090440	0.04705822	-0.0303064	1.23E-08	37	1	3099233	PRDM16;PRI	NM_022114;	Body;Body	chr1:310254	N_Shelf	NA	NA	NA	NA	1:3099210-3	Unclassified	NA	280232

cg02538891	-0.0578538	0.05753666	1.24E-08	37	9	139549426	NA	NA	NA	chr9:139551	N_Shore	NA	NA	NA	NA	NA	NA	NA	51714
cg13937758	-0.0845052	0.0886205	1.24E-08	37	6	150217448	NA	NA	NA	NA	NA	NA	NA	TRUE	NA	6:150217024	Unclassified_	TRUE	258085
cg16039660	0.05775249	-0.037094	1.26E-08	37	4	184686701	NA	NA	NA	NA	NA	NA	NA	NA	4:184923695	NA	NA	NA	295426
cg10363926	-0.0406944	0.0192488	1.30E-08	37	2	109921282	SH3RF3	NM_001099	Body	NA	NA	NA	NA	NA	NA	NA	NA	NA	197137
cg05525649	0.0465388	-0.0398587	1.30E-08	37	6	32156269	PBX2	NM_002586	Body	NA	NA	NA	NA	NA	NA	NA	NA	NA	109606
cg14522990	0.0429065	-0.0245976	1.36E-08	37	6	31691442	C6orf25;C6or	NM_138274;	Body;Body;Body;Body;B	chr6:316914	Island	NA	NA	NA	6:31799014-	NA	NA	NA	270175
cg23178192	0.03129531	-0.0147094	1.38E-08	37	16	28075295	GSG1L	NM_001109	TSS1500	chr16:28073	Island	NA	NA	NA	NA	16:28075032	Unclassified_	NA	408549
cg02343628	0.13548264	-0.0579835	1.39E-08	37	15	93790914	NA	NA	NA	NA	NA	NA	NA	TRUE	NA	NA	NA	NA	47875
cg11846580	0.06332917	-0.0392914	1.40E-08	37	16	88590409	ZFPM1	NM_153813	Body	chr16:88590	Island	NA	NA	NA	16:87117783	NA	NA	NA	222007
cg13992911	-0.0670575	0.06036901	1.46E-08	37	5	1003504	NA	NA	NA	chr5:100393	N_Shore	NA	CDMR	NA	NA	5:1003409-1	Unclassified_	TRUE	259200
cg10558233	-0.1399286	0.1523904	1.49E-08	37	8	94892613	NA	NA	NA	NA	NA	NA	NA	TRUE	NA	NA	NA	NA	200569
cg05701418	-0.1560773	0.15801089	1.50E-08	37	6	30131361	TRIM15;TRIN	NM_033229;	5'UTR;1stExon	NA	NA	NA	NA	TRUE	NA	NA	NA	NA	112716
cg07682160	0.06662021	-0.0391438	1.51E-08	37	19	18959935	UPF1	NM_002911	Body	NA	NA	NA	RDMR	NA	NA	NA	NA	TRUE	149664
cg14651616	0.06718063	-0.0672635	1.55E-08	37	11	64563992	MAP4K2	NM_004579	Body	NA	NA	NA	NA	NA	NA	NA	NA	NA	272843
cg14207833	0.04539462	-0.0434369	1.56E-08	37	19	18945153	UPF1	NM_002911	Body	chr19:18942	S_Shelf	NA	NA	TRUE	NA	NA	NA	NA	263748
cg20731257	0.05271475	-0.0460107	1.56E-08	37	2	87883497	NA	NA	NA	NA	NA	NA	NA	NA	2:87664612-	NA	NA	NA	371388
cg21350153	0.05670112	-0.051266	1.57E-08	37	2	95982371	KCNIP3	NM_013434	Body	NA	NA	NA	NA	TRUE	NA	2:95982334-	Unclassified_	TRUE	380796
cg12282552	0.05226323	-0.059337	1.58E-08	37	11	65321806	LTBP3;LTBP3	NM_001164	Body;Body;Body	chr11:65321	Island	NA	NA	NA	NA	NA	NA	NA	229295
cg03485217	-0.0702447	0.06972419	1.58E-08	37	4	10686300	CLNK;CLNK	NM_052964;	1stExon;5'UTR	NA	NA	NA	NA	NA	NA	NA	NA	NA	70465
cg14047370	-0.0974301	0.10807649	1.59E-08	37	7	43342206	HECW1	NM_015052	Body	NA	NA	NA	NA	TRUE	NA	NA	NA	TRUE	260439
cg14556762	-0.1118928	0.12931125	1.61E-08	37	1	170532470	NA	NA	NA	NA	NA	NA	NA	TRUE	NA	NA	NA	NA	270916
cg23419447	0.05119583	-0.0527206	1.61E-08	37	11	1507347	HCCA2	NM_053005	Body	NA	NA	NA	NA	NA	11:1463835-	NA	NA	TRUE	412670
cg13919908	-0.0739588	0.08823121	1.63E-08	37	11	33394273	NA	NA	NA	chr11:33397	N_Shelf	NA	NA	NA	NA	NA	NA	TRUE	257686
cg09225861	0.08753714	-0.0871243	1.65E-08	37	11	65069680	NA	NA	NA	NA	NA	NA	NA	TRUE	NA	NA	NA	TRUE	177174
cg26335760	0.06464216	-0.0465012	1.66E-08	37	3	133606960	RAB6B	NM_016577	Body	NA	NA	NA	NA	TRUE	NA	NA	NA	NA	459552
cg11203616	0.07354835	-0.0728031	1.67E-08	37	7	47576580	TNS3	NM_022748	5'UTR	chr7:475765	Island	NA	NA	NA	7:47542529-	NA	NA	TRUE	211224
cg10264003	-0.0554381	0.02105983	1.67E-08	37	11	110198965	NA	NA	NA	NA	NA	NA	NA	NA	NA	NA	NA	NA	195458
cg07141605	0.06887587	-0.0234234	1.67E-08	37	12	133005406	NA	NA	NA	NA	NA	NA	NA	NA	12:13151490	12:13300533	Unclassified_	NA	139584
cg12304937	-0.067285	0.06741447	1.68E-08	37	7	680013	PRKAR1B;PR	NM_001164	Body;Body;Body;Body;B	chr7:675569-	S_Shelf	NA	NA	NA	NA	NA	NA	NA	229656
cg20005742	0.07168415	-0.0621176	1.68E-08	37	14	103007070	NA	NA	NA	chr14:10301	N_Shelf	NA	NA	NA	NA	NA	NA	NA	360302
cg08137888	0.03326302	-0.015773	1.69E-08	37	20	825798	FAM110A;FA	NM_001042	Body;Body;1stExon	chr20:82526	Island	NA	NA	NA	20:773285-7	NA	NA	NA	157981
cg01129459	0.04836609	-0.0304956	1.72E-08	37	14	60386828	NA	NA	NA	chr14:60386	S_Shore	NA	NA	TRUE	14:59455991	14:60386651	Unclassified_	TRUE	23791
cg03546163	-0.1374814	0.13357315	1.77E-08	37	6	35654363	FKBP5;FKBP5	NM_004117;	5'UTR;5'UTR;5'UTR;5'U	chr6:356556	N_Shore	NA	NA	NA	NA	NA	NA	NA	71700
cg06853339	0.0839264	-0.0791833	1.83E-08	37	17	76117687	TMC6;TMC6	NM_001127	Body;Body	NA	NA	NA	NA	NA	17:73629267	17:76117019	Promoter_As	NA	133809
cg06636678	-0.0862212	0.07227036	1.86E-08	37	11	104576302	NA	NA	NA	NA	NA	NA	NA	TRUE	NA	11:10457615	Promoter_As	NA	129428
cg26995224	0.06544329	-0.0594805	1.89E-08	37	12	121974146	KDM2B;KDM	NM_032590;	Body;Body	chr12:12197	N_Shore	NA	RDMR	TRUE	12:12045839	12:12197407	Promoter_As	NA	471115
cg15586393	0.04637217	-0.0481739	1.90E-08	37	4	1244086	CTBP1;CTBP1	NM_001328;	TSS1500;TSS1500;TSS2	chr4:124141	Island	NA	NA	NA	NA	NA	NA	NA	287831
cg05992368	0.04365441	-0.0431201	1.92E-08	37	17	1572755	PRPF8	NM_006445	Body	NA	NA	NA	NA	TRUE	NA	17:1572650-	Gene_Associ	NA	118172
cg19755435	-0.116347	0.122658	1.93E-08	37	14	88473001	GPR65	NM_003608	5'UTR	NA	NA	NA	NA	NA	NA	14:88472565	Promoter_As	NA	356011
cg06601581	0.06150638	-0.0563289	1.93E-08	37	16	85404654	NA	NA	NA	chr16:85407	N_Shelf	NA	NA	TRUE	NA	NA	NA	NA	128691
cg17362247	0.05099091	-0.0482449	1.96E-08	37	19	39788647	IL29	NM_172140	Body	NA	NA	NA	NA	NA	NA	19:39788587	Unclassified_	NA	317814
cg01759628	0.05767771	-0.0460102	1.98E-08	37	1	235266653	NA	NA	NA	chr1:235267	N_Shore	NA	NA	NA	NA	NA	NA	NA	36012
cg03236948	0.06318906	-0.0417686	2.00E-08	37	11	63997492	DNAJC4	NM_005528	TSS1500	chr11:63997	N_Shore	NA	NA	NA	11:63754001	NA	NA	NA	65661
cg03393889	0.04368246	-0.049977	2.02E-08	37	16	2094700	NTHL1	NM_002528	Body	chr16:20974	N_Shelf	NA	NA	NA	16:2034603-	NA	NA	NA	68679
cg05253759	0.04791192	-0.0393928	2.06E-08	37	17	26941260	FLJ25006	NM_144610	TSS200	NA	NA	NA	NA	TRUE	NA	17:26939618	Gene_Associ	NA	104509
cg12233463	-0.1251786	0.13251749	2.10E-08	37	15	81679567	NA	NA	NA	NA	NA	NA	NA	TRUE	NA	NA	NA	NA	228587
cg05164926	0.09087968	-0.0342639	2.11E-08	37	17	7255624	KCTD11;KCTD	NM_001002	5'UTR;1stExon	chr17:72546	Island	high-CpG:71	NA	NA	17:7195142-	17:7253029-	Promoter_As	NA	102830
cg03173502	0.06158578	-0.0605576	2.14E-08	37	6	15505345	JARID2	NM_004973	Body	NA	NA	NA	NA	NA	6:15613324-	NA	NA	TRUE	64455
cg01798157	0.09810773	-0.1009299	2.17E-08	37	1	203276595	BTG2	NM_006763	3'UTR	chr1:203274	S_Shore	NA	NA	NA	NA	1:203276430	Promoter_As	NA	36819
cg09258479	0.03869873	-0.016428	2.18E-08	37	1	47655861	PDZK1IP1	NM_005764	TSS200	NA	NA	NA	NA	NA	NA	NA	NA	TRUE	177838
cg00591515	0.05288223	-0.0459071	2.19E-08	37	19	54971526	LENG8	NM_052925	Body	chr19:54971	N_Shore	NA	NA	NA	NA	NA	NA	NA	12648
cg09685472	-0.081531	0.09553974	2.20E-08	37	16	81527427	CMIP	NM_198390	Body	NA	NA	NA	NA	TRUE	16:80084844	16:81527395	Unclassified_	TRUE	185305
cg10980948	0.05331359	-0.056221	2.24E-08	37	17	80402614	C17orf62;C17	NM_001100	Body;Body;Body	chr17:80402	Island	NA	NA	NA	17:77994485	NA	NA	NA	207537
cg26840970	-0.0830626	0.07637787	2.25E-08	37	16	71523432	ZNF19	NM_006961	TSS200	NA	NA	NA	NA	NA	16:70080707	NA	NA	NA	468319
cg14260530	0.05341857	-0.0495516	2.26E-08	37	9	117157871	AKNA	NM_030767	TSS1500	chr9:117160	N_Shelf	NA	NA	NA	NA	NA	NA	NA	264915
cg02056751	0.05281141	-0.0539745	2.30E-08	37	11	2320647	C11orf21;C11	NR_024621;	Body;3'UTR	NA	NA	NA	NA	NA	NA	11:2320408-	Unclassified_	TRUE	42153
cg00853733	0.08602293	-0.0945293	2.30E-08	37	12	122244223	SETD1B	NM_015048	Body	chr12:12224	S_Shore	NA	RDMR	NA	NA	12:12224406	Promoter_As	NA	17979
cg08324862	-0.0868428	0.07473822	2.37E-08	37	12	77800655	NA	NA	NA	NA	NA	NA	NA	TRUE	NA	NA	NA	NA	161538
cg05122026	0.04821969	-0.0453393	2.39E-08	37	11	75114223	SNORD15B;F	NR_000025;	TSS1500;Body	chr11:75110	S_Shelf	NA	NA	NA	NA	NA	NA	NA	101963
cg15696506	-0.0863441	0.10300269	2.40E-08	37	4	57947735	IGFBP7	NM_001553	Body	NA	NA	NA	NA	TRUE	NA	NA	NA	TRUE	289747
cg10816169	0.0534987	-0.0466341	2.40E-08	37	11	66080868	NA	NA	NA	chr11:66079	Island	NA	NA	NA	11:65835972	11:66078582	Promoter_As	NA	204854
cg19570155	0.0796953	-0.0746675	2.46E-08	37	2	36765605	CRIM1	NM_016441	Body	NA	NA	NA	NA	TRUE	NA	NA	NA	NA	352781
cg23461714	0.04821157	-0.0356474	2.54E-08	37	11	113184990	TTC12	NM_017868	TSS1500	chr11:11318	N_Shore	NA	NA	NA	NA	11:11318483	Promoter_As	NA	413266
cg04952324	0.04551174	-0.0222722	2.56E-08	37	7	73727108	CLIP2;CLIP2	NM_032421;	5'UTR;5'UTR	NA	NA	NA	NA	TRUE	NA	NA	NA	TRUE	98825
cg26196424	-0.0608063	0.05314855	2.64E-08	37	14	58615960	C14orf37	NM_001001	5'UTR	chr14:58618	N_Shelf	NA	NA	NA	NA	NA	NA	NA	457100
cg06669226	0.05363808	-0.0288391	2.65E-08	37	7	72713786	NA	NA	NA	chr7:727116	S_Shore	NA	NA	NA	NA	NA	NA	NA	130067
cg12385729	0.05804504	-0.057793	2.73E-08	37	1	9243679	NA	NA	NA	chr1:924216	Island	NA	NA	NA	1:9164646-9	NA	NA	NA	230711

cg03726147	0.03972996	-0.03474	2.74E-08	37	1	44686022	DMAP1;DMA	NM_019100;	Body;Body;Body	NA	NA	NA	NA	TRUE	1:44458547-	1:44685614-	Gene_Associ	NA	75286
cg07461273	0.05980954	-0.0640734	2.79E-08	37	7	99697172	MCM7;MCM	NM_005916;	Body;5'UTR	chr7:996981	N_Shore	NA	NA	NA	7:99535108-	NA	NA	NA	145498
cg26928195	0.05675409	-0.0514457	2.81E-08	37	19	1624734	TCF3;TCF3	NM_003200;	Body;Body	chr19:16253	N_Shore	NA	NA	NA	19:1575448-	19:1624446-	Gene_Associ	TRUE	469898
cg04968127	0.07071385	-0.066466	2.84E-08	37	11	10715213	MRVI1;MRV	NM_00110	1stExon;5'UTR;1stExon	NA	NA	low-CpG:106	NA	TRUE	NA	NA	NA	NA	99102
cg19295068	0.06620466	-0.0509753	2.88E-08	37	19	48206119	GLTSCR1	NM_015711	3'UTR	chr19:48204	S_Shore	NA	NA	NA	19:52896368	NA	NA	NA	348448
cg26376241	-0.1093188	0.14522179	2.94E-08	37	2	65594021	SPRED2;SPR	NM_001128	TSS200;Body	NA	NA	NA	NA	TRUE	NA	NA	NA	TRUE	460318
cg12716083	0.05093774	-0.0441463	2.96E-08	37	6	30559087	ABCF1;ABCF	NM_001090;	3'UTR;3'UTR	NA	NA	NA	NA	TRUE	NA	NA	NA	TRUE	236175
cg12155969	0.05664478	-0.0331491	2.97E-08	37	15	92849000	NA	NA	NA	NA	NA	NA	NA	TRUE	NA	NA	NA	NA	227282
cg25730428	0.05289149	-0.0326714	3.04E-08	37	6	29454755	MAS1L	NM_052967	1stExon	NA	NA	NA	NA	NA	NA	NA	NA	NA	449732
cg23051123	0.03739626	-0.02955	3.09E-08	37	1	226132841	NA	NA	NA	NA	NA	NA	NA	TRUE	NA	1:226132766	Unclassified_	NA	406503
cg24136318	0.05314018	-0.0613718	3.09E-08	37	17	75454130	SEPT9;SEPT9	NM_001113	Body;5'UTR;Body;Body	NA	NA	NA	NA	TRUE	NA	NA	NA	NA	424317
cg26169081	0.06343801	-0.0722517	3.11E-08	37	10	12648338	CAMK1D;CAN	NM_020397;	Body;Body	NA	NA	NA	NA	TRUE	10:12688103	10:12647615	Promoter_As	NA	456656
cg26929394	-0.0653326	0.04933147	3.13E-08	37	11	112435198	NA	NA	NA	NA	NA	NA	NA	TRUE	NA	NA	NA	NA	469925
cg17652998	0.04225228	-0.03705	3.15E-08	37	10	101370592	SLC25A28	NM_031212	3'UTR	NA	NA	NA	NA	NA	NA	NA	NA	NA	322340
cg05242915	0.0736463	-0.0646129	3.16E-08	37	19	1263080	NA	NA	NA	chr19:12602	S_Shore	NA	DMR	NA	NA	NA	NA	NA	104329
cg05969150	0.03593008	-0.0216793	3.16E-08	37	16	72882495	ZFHX3;ZFHX	NM_001164	Body;Body	NA	NA	NA	NA	TRUE	NA	NA	NA	TRUE	117686
cg03125341	0.05031465	-0.0312543	3.23E-08	37	6	157882207	ZDHHC14;ZD	NM_153746;	Body;Body	NA	NA	NA	NA	TRUE	NA	6:157882056	Unclassified	NA	63504
cg19087971	0.06536632	-0.0597797	3.23E-08	37	7	751233	PRKAR1B;PR	NM_002735;	5'UTR;5'UTR;5'UTR;5'U	chr7:750788-	Island	NA	NA	NA	7:717343-71	NA	NA	TRUE	345403
cg19838043	0.05751016	-0.05664	3.24E-08	37	14	104196038	ZFYVE21	NM_024071	Body	chr14:10419	S_Shore	NA	NA	NA	14:10326563	14:10419598	Gene_Associ	TRUE	357457
cg18073471	0.08611639	-0.0659923	3.28E-08	37	4	81119198	PRDM8;PRD	NM_020226;	5'UTR;5'UTR	chr4:811190	Island	NA	NA	NA	4:81338101-	4:81118358-	Unclassified_	NA	328986
cg17439800	0.06974562	-0.0658323	3.28E-08	37	1	208056493	NA	NA	NA	NA	NA	NA	NA	NA	NA	1:208056036	Promoter_As	NA	319069
cg09546258	-0.0753792	0.08644381	3.31E-08	37	11	68444995	NA	NA	NA	NA	NA	NA	NA	TRUE	NA	NA	NA	TRUE	182892
cg20587874	-0.087453	0.11706553	3.32E-08	37	2	179269232	MIR548N	NR_031666	Body	NA	NA	NA	NA	TRUE	NA	2:179269210	Unclassified_	NA	368967
cg05673882	-0.0668438	0.07855897	3.33E-08	37	5	74862702	POLK	NM_016218	Body	NA	NA	NA	NA	TRUE	NA	NA	NA	NA	112278
cg02512902	-0.0977855	0.07117783	3.36E-08	37	17	25799447	KSR1	NM_014238	5'UTR	NA	NA	NA	NA	TRUE	NA	NA	NA	NA	51195
cg00294382	0.05993509	-0.0434013	3.37E-08	37	12	56732721	IL23A;IL23A	NM_016584;	5'UTR;1stExon	NA	NA	NA	NA	TRUE	NA	12:56732294	Promoter_As	NA	6197
cg26790897	0.07776493	-0.0626262	3.38E-08	37	7	56147772	SUMF2;SUM	NM_001042	3'UTR;3'UTR;3'UTR;3'U	NA	NA	NA	NA	TRUE	NA	7:56146865-	Gene_Associ	NA	467320
cg13522406	0.07160924	-0.0517192	3.39E-08	37	6	30581820	PPP1R10	NM_002714	5'UTR	chr6:305848	N_Shelf	NA	NA	NA	NA	NA	NA	NA	249516
cg17038235	0.07326067	-0.0485955	3.42E-08	37	5	172370524	ERGIC1	NM_001031	Body	NA	NA	NA	NA	TRUE	NA	NA	NA	TRUE	312466
cg15251319	0.04272655	-0.043615	3.42E-08	37	3	194836302	C3orf21	NM_152531	Body	NA	NA	NA	NA	NA	3:196317591	NA	NA	NA	282765
cg07183799	0.0476577	-0.0531142	3.48E-08	37	13	107214285	ARGLU1	NM_018011	Body	NA	NA	NA	NA	TRUE	NA	NA	NA	NA	140520
cg09085220	0.03445511	-0.0251791	3.49E-08	37	16	8736433	C16orf68	NM_024109	Body	NA	NA	NA	NA	NA	16:8643828-	NA	NA	NA	174820
cg00382138	-0.1302134	0.17587277	3.50E-08	37	4	110723299	CFI;CFI	NM_000204;	1stExon;5'UTR	NA	NA	low-CpG:110	NA	NA	NA	NA	NA	NA	8086
cg26550214	-0.1388842	0.11679721	3.58E-08	37	5	34609493	NA	NA	NA	NA	NA	NA	NA	TRUE	NA	NA	NA	TRUE	463314
cg20100049	0.05846516	-0.0599712	3.59E-08	37	11	67979188	SUV420H1;S	NM_017635;	5'UTR;5'UTR	chr11:67980	N_Shore	NA	NA	NA	11:67735641	11:67978656	Promoter_As	NA	361719
cg07699845	0.06208977	-0.0336059	3.59E-08	37	8	38068205	BAG4	NM_004874	3'UTR	NA	NA	NA	NA	NA	NA	NA	NA	NA	149984
cg25207828	0.04412639	-0.0258098	3.68E-08	37	3	9791044	OGG1;OGG1	NM_016828;	TSS1500;TSS1500;TSS1	chr3:979112	N_Shore	NA	NA	NA	NA	3:9790917-9	Promoter_As	NA	441619
cg25410739	0.0409188	-0.0234853	3.73E-08	37	1	223743086	CAPN8	NM_001143	Body	chr1:223741	Island	NA	NA	NA	1:221808589	NA	NA	NA	444806
cg09567048	-0.0490802	0.03366214	3.77E-08	37	2	46456880	NA	NA	NA	NA	NA	NA	NA	TRUE	NA	2:46456844-	Unclassified_	NA	183306
cg27395208	0.03115919	-0.0148301	3.80E-08	37	11	128557481	NA	NA	NA	chr11:12856	N_Shelf	NA	RDMR	NA	11:12806245	NA	NA	TRUE	477868
cg20605134	0.0431017	-0.011442	3.85E-08	37	6	15400462	JARID2	NM_004973	Body	NA	NA	NA	RDMR	TRUE	NA	NA	NA	TRUE	369233
cg03254465	0.08576125	-0.0521348	3.87E-08	37	1	3240227	PRDM16;PRD	NM_022114;	Body;Body	chr1:323991	Island	NA	NA	NA	1:3229442-3	NA	NA	NA	65971
cg24074477	0.06850977	-0.0437392	3.89E-08	37	22	17956455	CECR2	NM_031413	TSS200	NA	NA	NA	NA	NA	NA	NA	NA	NA	423231
cg14702960	-0.0427359	0.03058362	3.89E-08	37	12	68634040	NA	NA	NA	NA	NA	NA	NA	TRUE	NA	NA	NA	TRUE	273742
cg16903269	-0.0556645	0.03440794	3.93E-08	37	7	134250389	AKR1B15	NM_001080	Body	NA	NA	NA	NA	NA	NA	NA	NA	NA	310381
cg01101873	0.05801601	-0.0376201	3.93E-08	37	1	3269478	PRDM16;PRD	NM_022114;	Body;Body	NA	NA	NA	NA	TRUE	1:3259175-3	NA	NA	TRUE	23259
cg10437265	0.03398728	-0.0126757	3.97E-08	37	15	77819839	NA	NA	NA	NA	NA	NA	NA	TRUE	NA	NA	NA	NA	198328
cg02916283	-0.0762554	0.06711148	4.00E-08	37	14	90087792	NA	NA	NA	chr14:90085	S_Shelf	NA	NA	NA	NA	14:90087339	Unclassified_	NA	59322
cg07835482	-0.1596282	0.17224721	4.10E-08	37	2	182656091	NA	NA	NA	NA	NA	NA	NA	TRUE	NA	NA	NA	NA	152502
cg21618521	0.06884673	-0.0611936	4.12E-08	37	6	33245770	B3GALT4	NM_003782	1stExon	chr6:332446	S_Shore	NA	NA	TRUE	6:33352687-	6:33244505-	Promoter_As	NA	384872
cg17366225	-0.0855571	0.10789046	4.28E-08	37	4	11398983	NA	NA	NA	chr4:114007	N_Shore	NA	NA	NA	NA	NA	NA	NA	317865
cg26351132	-0.0555069	0.05191611	4.30E-08	37	17	47049890	NA	NA	NA	NA	NA	NA	DMR	TRUE	NA	NA	NA	TRUE	459862
cg11077681	0.07219768	-0.0544564	4.34E-08	37	11	10715188	MRVI1;MRV	NM_00110	1stExon;5'UTR;1stExon	NA	NA	low-CpG:106	NA	TRUE	NA	NA	NA	NA	209173
cg05517572	0.05190296	-0.0496782	4.34E-08	37	19	4338769	STAP2;STAP	NM_001013	1stExon;5'UTR;5'UTR;1	NA	NA	NA	NA	NA	NA	NA	NA	TRUE	109474
cg12656312	-0.0742123	0.04312126	4.39E-08	37	11	6024266	OR56A4	NM_001005	1stExon	NA	NA	NA	NA	NA	NA	NA	NA	NA	235307
cg10038867	-0.0410298	0.04559903	4.39E-08	37	1	21982511	RAP1GAP;RA	NM_002885;	5'UTR;5'UTR	chr1:219857	N_Shelf	NA	NA	NA	NA	NA	NA	TRUE	191296
cg26657404	0.06015484	-0.0885157	4.42E-08	37	16	85684821	KIAA0182;KI	NM_001134	Body;Body	chr16:85684	S_Shore	NA	NA	NA	16:84241637	16:85684742	Unclassified	NA	465073
cg16672223	0.03776147	-0.0237401	4.45E-08	37	16	81650677	CMIP;CMIP	NM_030629;	Body;Body	NA	NA	NA	NA	TRUE	NA	16:81649729	Promoter_As	TRUE	306563
cg02218324	0.05209913	-0.0374205	4.47E-08	37	19	46318439	RSPH6A;RSP	NM_030785;	1stExon;5'UTR	chr19:46318	N_Shore	NA	NA	NA	19:51010222	NA	NA	NA	45287
cg26729204	0.04759119	-0.0568786	4.72E-08	37	6	2932241	NA	NA	NA	NA	NA	NA	NA	TRUE	NA	6:2931511-2	Unclassified	NA	466390
cg06653796	0.06608232	-0.0817813	4.79E-08	37	20	62367805	LIME1	NM_017806	TSS200	chr20:62368	N_Shore	NA	CDMR	NA	NA	20:62367587	Promoter_As	NA	129796
cg21348975	-0.0540318	0.03648511	4.82E-08	37	12	48724119	NA	NA	NA	chr12:48723	S_Shore	NA	NA	NA	NA	NA	NA	NA	380778
cg04119529	0.04517609	-0.0428413	4.85E-08	37	16	2094667	NTHL1	NM_002528	Body	chr16:20974	N_Shelf	NA	NA	NA	16:2034603-	NA	NA	NA	82758
cg22959742	0.08656515	-0.0594792	4.90E-08	37	10	13913931	FRMD4A	NM_018027	Body	NA	NA	NA	NA	TRUE	NA	NA	NA	NA	404921
cg01904812	0.0958291	-0.0815951	4.93E-08	37	1	17746340	RCC2;RCC2	NM_018715;	Body;Body	chr1:177462	Island	NA	NA	NA	1:17618753-	1:17745852-	Gene_Associ	NA	39003
cg26979518	-0.0385533	0.02827957	4.95E-08	37	12	56078391	ITGA7;ITGA7	NM_001144	3'UTR;3'UTR;3'UTR;3'U	NA	NA	NA	NA	NA	NA	NA	NA	NA	470817
cg13628022	-0.0913228	0.06618474	4.99E-08	37	9	124726047	TTLL11	NM_001139	Body	NA	NA	NA	NA	TRUE	NA	NA	NA	NA	251765

cg12804791	0.07783942	-0.0536644	5.02E-08	37	11	126286828	NA	NA	NA	chr11:12628	Island	NA	DMR	TRUE	11:12579168	NA	NA	TRUE	237513
cg19548859	0.09384672	-0.0510315	5.03E-08	37	10	11239482	CUGBP2;CU	NM_001025	Body;Body;Body;Body	NA	NA	NA	NA	TRUE	NA	10:11239247	Promoter_As	NA	352475
cg25299364	-0.0565199	0.05526522	5.04E-08	37	2	68882144	PROKR1	NM_138964	Body	chr2:688823	N_Shore	NA	NA	NA	2:68735621-	NA	NA	NA	442921
cg04347477	0.05358106	-0.0187845	5.05E-08	37	12	125002007	NCOR2;NCO	NM_006312;	5'UTR;5'UTR	chr12:12500	Island	NA	NA	NA	12:12356792	NA	NA	NA	87306
cg10000764	-0.1091756	0.07425724	5.05E-08	37	7	129494118	UBE2H;UBE2	NM_003344;	Body;Body	NA	NA	NA	NA	TRUE	NA	NA	NA	NA	190635
cg24859236	0.05443794	-0.049271	5.08E-08	37	1	9750213	PIK3CD	NM_005026	5'UTR	chr1:974929	Island	NA	NA	NA	1:9671695-9	1:9748906-9	Unclassified	NA	436007
cg01368075	0.05577054	-0.0497962	5.10E-08	37	3	128523902	RAB7A	NM_004637	Body	NA	NA	NA	NA	TRUE	NA	NA	NA	NA	28435
cg21408813	-0.1512158	0.13167143	5.27E-08	37	13	52736798	NA	NA	NA	chr13:52733	S_Shelf	NA	NA	NA	NA	NA	NA	TRUE	381528
cg12170787	-0.1114461	0.10188836	5.29E-08	37	19	1130965	SBNO2;SBN	NM_001100	Body;Body	NA	NA	NA	DMR	NA	NA	19:1130697-	Promoter_As	NA	227555
cg10501360	0.06313828	-0.0516397	5.31E-08	37	10	49671665	ARHGAP22	NM_021226	Body	chr10:49674	N_Shelf	NA	NA	TRUE	NA	NA	NA	NA	199436
cg26172016	-0.0567299	0.02831319	5.34E-08	37	18	56529149	ZNF532	NM_018181	TSS1500	chr18:56530	N_Shore	NA	RDMR	NA	NA	18:56529108	Unclassified_	NA	456704
cg11216632	0.03494973	-0.0219435	5.37E-08	37	1	155944655	ARHGEF2;AR	NM_001162	Body;Body;5'UTR	chr1:155947	N_Shelf	NA	NA	NA	NA	1:155944541	Promoter_As	NA	211459
cg14471064	0.0368553	-0.0278381	5.50E-08	37	9	139236663	GPSM1	NM_001145	Body	chr9:139236	N_Shore	NA	NA	NA	NA	9:139236401	Unclassified	NA	269071
cg05036937	-0.0426254	0.03283904	5.58E-08	37	5	52283760	ITGA2	NM_002203	TSS1500	chr5:522847	N_Shore	NA	NA	NA	NA	NA	NA	NA	100423
cg07452706	0.04112467	-0.0189248	5.65E-08	37	14	105649097	NUDT14	NM_177533	TSS1500	chr14:10564	S_Shore	NA	NA	NA	NA	14:10564888	Unclassified_	NA	145340
cg00472758	0.08660148	-0.0831749	5.67E-08	37	16	2552820	TBC1D24	NM_020705	3'UTR	chr16:25500	S_Shelf	NA	NA	TRUE	NA	16:2552796-	Unclassified_	NA	10014
cg06469726	-0.0739571	0.05724217	5.70E-08	37	2	242483467	NA	NA	NA	chr2:242480	S_Shore	NA	NA	NA	NA	NA	NA	TRUE	126290
cg20458044	-0.0444032	0.03802798	5.70E-08	37	17	57904327	TMEM49	NM_030938	Body	NA	NA	NA	NA	TRUE	NA	NA	NA	NA	367033
cg18394552	0.08568754	-0.0639249	5.73E-08	37	5	159428643	NA	NA	NA	NA	NA	NA	NA	TRUE	NA	NA	NA	NA	334402
cg05500734	-0.0683784	0.0841114	5.75E-08	37	7	73437649	NA	NA	NA	NA	NA	NA	NA	TRUE	NA	7:73437299-	Unclassified_	TRUE	109124
cg03345925	0.05479995	-0.0649302	5.80E-08	37	8	144599347	ZC3H3	NM_015117	Body	chr8:144601	N_Shelf	NA	NA	NA	8:144670116	8:144598000	Unclassified_	NA	67771
cg02879453	0.08522057	-0.0855938	5.81E-08	37	16	50321818	ADCY7	NM_001114	TSS200	NA	NA	NA	NA	NA	NA	NA	NA	NA	58518
cg27526649	0.05158892	-0.0307938	5.81E-08	37	1	208132004	NA	NA	NA	chr1:208132	N_Shore	NA	NA	NA	NA	1:208131746	Unclassified_	NA	479997
cg17507897	-0.0492483	0.02163227	5.94E-08	37	20	17943694	SNX5;SNORD	NM_014426;	Body;TSS200;Body	NA	NA	NA	NA	NA	NA	NA	NA	NA	320236
cg22729726	0.040488	-0.0219711	5.98E-08	37	1	3123854	PRDM16;PRD	NM_022114;	Body;Body	NA	NA	NA	NA	NA	1:3113664-3	NA	NA	NA	401161
cg13578465	0.06947786	-0.074835	6.03E-08	37	14	105167457	INF2;INF2;IN	NM_022489;	5'UTR;5'UTR;5'UTR	chr14:10516	Island	high-CpG:10	NA	NA	14:10423832	NA	NA	NA	250731
cg23464284	0.09553962	-0.0821277	6.07E-08	37	6	166996837	RPS6KA2;RP	NM_021135;	Body;Body	NA	NA	NA	NA	TRUE	NA	NA	NA	NA	413319
cg18833140	-0.0410844	0.03378692	6.08E-08	37	10	115312553	HABP2	NM_004132	TSS1500	NA	NA	NA	NA	NA	NA	NA	NA	NA	341599
cg11155784	0.0330613	-0.0058554	6.12E-08	37	1	164572163	PBX1	NM_002585	Body	NA	NA	NA	NA	TRUE	NA	NA	NA	NA	210423
cg24843346	0.06336886	-0.058543	6.13E-08	37	8	142736091	NA	NA	NA	chr8:142734	S_Shore	NA	NA	NA	NA	NA	NA	NA	435720
cg25072766	0.08397706	-0.0733384	6.21E-08	37	11	3073488	CARS;CARS;	NM_001014	Body;Body;5'UTR;Body	NA	NA	NA	NA	NA	11:3029984-	NA	NA	NA	439518
cg05573550	0.04576104	-0.0256694	6.29E-08	37	4	148890890	ARHGAP10	NM_024605	Body	NA	NA	NA	NA	TRUE	NA	NA	NA	NA	110404
cg01839993	0.04031678	-0.0460775	6.38E-08	37	10	74034644	DDIT4	NM_019058	Body	chr10:74033	Island	NA	NA	NA	10:73703174	10:74034192	Promoter_As	NA	37786
cg10369363	0.04437302	-0.021023	6.46E-08	37	12	52404422	GRASP	NM_181711	Body	chr12:52408	N_Shelf	NA	NA	TRUE	NA	12:52403987	Unclassified_	TRUE	197237
cg01400671	0.05812753	-0.04422	6.55E-08	37	9	90622100	NA	NA	NA	chr9:906215	Island	NA	NA	NA	9:89811409-	NA	NA	NA	29099
cg09645475	0.04184801	-0.0386436	6.57E-08	37	20	815316	FAM110A;FA	NM_207121;	TSS1500;5'UTR	chr20:81412	S_Shore	NA	NA	NA	NA	20:813724-8	Promoter_As	NA	184610
cg19719478	-0.0644918	0.05499803	6.65E-08	37	12	13529218	C12orf36	NM_182558	Body	NA	NA	NA	NA	NA	NA	NA	NA	NA	355306
cg16127514	-0.0584894	0.05022455	6.69E-08	37	10	29273678	NA	NA	NA	NA	NA	NA	NA	TRUE	NA	NA	NA	TRUE	296832
cg10119082	0.08361486	-0.0661949	6.73E-08	37	7	98990626	ARPC1B	NM_005720	Body	chr7:989901	Island	NA	NA	TRUE	7:98828146-	7:98990227-	Promoter_As	TRUE	192776
cg18923051	0.0397081	-0.0272	6.78E-08	37	1	156053080	MEX3A	NM_001093	TSS1500	chr1:156051	S_Shore	NA	NA	NA	NA	NA	NA	NA	343025
cg13213009	-0.1176743	0.10366464	6.79E-08	37	8	74903761	LY96	NM_015364	1stExon	NA	NA	NA	NA	TRUE	NA	NA	NA	NA	243693
cg07815238	-0.0350322	0.03255093	6.81E-08	37	15	64341005	NA	NA	NA	chr15:64338	S_Shelf	NA	NA	NA	NA	NA	NA	NA	152117
cg18042806	-0.0498538	0.04318544	6.85E-08	37	5	177659971	AGXT2L2	NM_153373	TSS200	chr5:177659	S_Shore	NA	NA	NA	NA	5:177658792	Promoter_As	NA	328465
cg14470121	0.04304037	-0.0373973	6.87E-08	37	16	1587684	IFT140;TMEI	NM_014714;	Body;Body	chr16:15876	Island	NA	NA	NA	16:1527106-	16:1587536-	Unclassified_	NA	269051
cg18485720	0.04161333	-0.0222644	6.91E-08	37	19	720740	PALM;PALM	NM_002579;	Body;Body	chr19:71909	Island	NA	NA	NA	19:670014-6	19:720678-7	Unclassified_	NA	336026
cg03316098	0.02618801	-0.0022561	6.99E-08	37	6	29601398	GABBR1;GA	NM_001470;	TSS1500;TSS1500	chr6:296001	S_Shore	NA	NA	NA	NA	6:29601275-	Promoter_As	NA	67149
cg08371947	0.05042274	-0.0469614	7.01E-08	37	4	8477793	C4orf23	NM_152544	3'UTR	NA	NA	NA	NA	NA	4:8528422-8	NA	NA	NA	162338
cg11317199	0.07783064	-0.0743709	7.08E-08	37	9	100850391	TRIM14;TRI	NM_014788;	Body;Body;Body;Body	chr9:100849	Island	NA	NA	NA	9:99889558-	NA	NA	NA	213170
cg14875081	0.03032039	-0.0248111	7.26E-08	37	2	96814660	NA	NA	NA	chr2:968104	S_Shelf	NA	DMR	NA	NA	2:96813678-	Promoter_As	TRUE	276638
cg09975715	0.05736856	-0.0545631	7.32E-08	37	17	8379050	MYH10	NM_005964	3'UTR	chr17:83801	N_Shore	NA	NA	NA	17:8319758-	NA	NA	NA	190199
cg05379350	0.04261779	-0.0318504	7.33E-08	37	17	27917157	GIT1;GIT1	NM_014030;	TSS1500;TSS1500	chr17:27918	N_Shore	NA	NA	NA	17:24939942	NA	NA	NA	107026
cg09696044	0.07115614	-0.0817832	7.33E-08	37	20	55968294	RBM38;RBM	NM_017495;	Body;Body	chr20:55966	S_Shore	NA	CDMR	TRUE	NA	20:55968089	Promoter_As	NA	185474
cg16204717	-0.0582389	0.04955151	7.35E-08	37	12	65894458	NA	NA	NA	NA	NA	NA	NA	TRUE	NA	NA	NA	NA	298084
cg02326058	0.08541132	-0.0639368	7.40E-08	37	8	8993102	NA	NA	NA	NA	NA	NA	NA	TRUE	NA	NA	NA	NA	47477
cg05360714	0.06945276	-0.0403501	7.45E-08	37	17	75543046	NA	NA	NA	NA	NA	low-CpG:730	NA	NA	NA	NA	NA	NA	106593
cg15697019	0.05765846	-0.0568832	7.48E-08	37	7	77357984	RSBN1L	NM_198467	Body	NA	NA	NA	NA	TRUE	NA	NA	NA	NA	289757
cg19598514	0.03930879	-0.0350199	7.50E-08	37	11	442149	ANO9	NM_001012	TSS200	NA	NA	NA	NA	TRUE	NA	11:441345-4	Unclassified	TRUE	353304
cg05372727	-0.0577905	0.06012988	7.56E-08	37	13	107029018	NA	NA	NA	NA	NA	NA	NA	NA	NA	13:10702848	Unclassified_	NA	106869
cg21934230	0.07329382	-0.053646	7.57E-08	37	1	228202608	WNT3A	NM_033131	Body	NA	NA	NA	NA	TRUE	NA	NA	NA	TRUE	389571
cg04368724	0.04400691	-0.0393473	7.63E-08	37	6	31760593	VARS	NM_006295	Body	chr6:317632	N_Shelf	NA	NA	NA	NA	NA	NA	NA	87707
cg10234998	0.04331226	-0.0168772	7.71E-08	37	6	29601491	GABBR1;GA	NM_001470;	TSS1500;TSS1500	chr6:296001	S_Shore	NA	NA	NA	NA	NA	NA	NA	194951
cg12061531	-0.0456035	0.03226644	7.73E-08	37	17	21729912	NA	NA	NA	chr17:21729	Island	NA	NA	NA	17:21653914	NA	NA	NA	225605
cg18643093	0.05059288	-0.0597164	7.82E-08	37	5	150521257	ANXA6;ANX	NM_001155;	Body;Body	NA	NA	NA	NA	TRUE	NA	5:150521159	Unclassified_	NA	338512
cg23534245	-0.0377926	0.0291447	7.84E-08	37	20	6751435	BMP2	NM_001200	Body	chr20:67480	S_Shore	NA	CDMR	NA	NA	NA	NA	NA	414515
cg23459424	-0.0922728	0.10663895	7.91E-08	37	2	28549125	BRE;BRE;BR	NM_199193;	3'UTR;Body;Body;Body	NA	NA	NA	NA	TRUE	NA	NA	NA	NA	413230
cg05183538	0.07418544	-0.0510809	8.02E-08	37	16	88536950	ZFPM1	NM_153813	Body	chr16:88536	Island	NA	NA	NA	NA	16:88536615	Promoter_As	NA	103174
cg19778462	0.03036325	-0.0085723	8.15E-08	37	1	41976072	HIVEP3;HIVE	NM_001127	3'UTR;3'UTR	NA	NA	NA	NA	NA	NA	NA	NA	NA	356459

cg06193043	0.05959781	-0.0505544	8.17E-08	37	1	11908199	NPPA	NM_006172	TSS1500	NA	NA	NA	NA	TRUE	NA	NA	NA	NA	121633
cg22509164	0.02749047	-0.0166816	8.22E-08	37	1	46766957	LRRC41	NM_006369	Body	chr1:467674	N_Shore	NA	RDMR	NA	NA	NA	NA	NA	397748
cg02454476	-0.0357866	0.02160328	8.23E-08	37	15	68844912	NA	NA	NA	NA	NA	NA	NA	TRUE	NA	NA	NA	NA	49958
cg25499181	0.05533784	-0.0288711	8.29E-08	37	10	30894357	NA	NA	NA	NA	NA	NA	NA	TRUE	NA	NA	NA	TRUE	446182
cg01054110	0.06479094	-0.0465536	8.33E-08	37	12	125002332	NCOR2;NCOI	NM_006312;	5'UTR;5'UTR	chr12:12500	Island	NA	NA	NA	12:12356792	NA	NA	NA	22208
cg07626482	-0.0321285	0.02273549	8.35E-08	37	19	47289503	SLC1A5;SLC1	NM_001145	TSS1500;Body;5'UTR	chr19:47290	N_Shore	NA	RDMR	NA	NA	NA	NA	NA	148720
cg19744498	0.0645633	-0.0561245	8.38E-08	37	2	208631081	FZD5	NM_003468	3'UTR	chr2:208631	N_Shore	NA	RDMR	NA	NA	NA	NA	NA	355810
cg18437480	0.0390705	-0.0534128	8.39E-08	37	17	76130305	TMC8	NM_152468	Body	chr17:76127	S_Shore	NA	NA	NA	17:73641728	17:76129950	Promoter_As	TRUE	335153
cg16589830	0.08886424	-0.0551971	8.43E-08	37	1	212284214	NA	NA	NA	NA	NA	NA	NA	TRUE	NA	NA	NA	NA	305057
cg00290023	-0.0341959	0.04294223	8.45E-08	37	5	174962968	NA	NA	NA	NA	NA	NA	NA	TRUE	NA	NA	NA	NA	6094
cg00136405	-0.0527359	0.03444912	8.57E-08	37	4	123542198	IL21;IL21	NM_021803;	5'UTR;1stExon	NA	NA	NA	NA	NA	NA	NA	NA	NA	3088
cg03147185	-0.0427911	0.03794551	8.62E-08	37	2	97008030	NCAPH	NM_015341	Body	NA	NA	NA	NA	TRUE	NA	2:97007871-	Unclassified	NA	63943
cg08512353	0.0460539	-0.0268493	8.74E-08	37	11	134230036	GLB1L2	NM_138342	Body	chr11:13423	N_Shore	NA	NA	NA	11:13373524	11:13422985	Unclassified_	NA	164791
cg20462883	0.03462113	-0.0254741	8.90E-08	37	12	122461621	BCL7A;BCL7A	NM_001024	Body;Body	NA	NA	NA	RDMR	TRUE	12:12094318	12:12246103	Unclassified	NA	367124
cg17416730	0.05928057	-0.0600926	9.25E-08	37	6	33245541	B3GALT4	NM_003782	1stExon	chr6:332446	Island	NA	NA	TRUE	6:33352687-	6:33244505-	Promoter_As	TRUE	318688
cg16764781	0.17736317	-0.1282956	9.31E-08	37	3	189347820	TP63;TP63;TI	NM_001114	TSS1500;TSS1500;TSS1	NA	NA	NA	NA	NA	NA	NA	NA	NA	308282
cg25542319	0.07353082	-0.0485267	9.41E-08	37	5	112540429	MCC;MCC	NM_002387;	Body;Body	NA	NA	NA	NA	TRUE	NA	5:112540314	Unclassified_	NA	446800
cg26182406	-0.1331246	0.16433185	9.41E-08	37	14	105491309	NA	NA	NA	chr14:10548	S_Shelf	low-CpG:104	NA	NA	NA	14:10549121	Unclassified	TRUE	456866
cg22790839	-0.0955114	0.0952336	9.44E-08	37	5	157883933	NA	NA	NA	NA	NA	NA	NA	TRUE	NA	NA	NA	TRUE	402015
cg21741998	-0.0874003	0.074558	9.46E-08	37	6	36788637	CPNE5	NM_020939	Body	NA	NA	NA	NA	TRUE	NA	NA	NA	NA	386539
cg23659289	0.04596797	-0.0177813	9.48E-08	37	17	43472725	ARHGAP27;A	NM_199282;	3'UTR;3'UTR	chr17:43472	Island	NA	NA	TRUE	17:40828323	17:43472209	Gene_Associ	NA	416463
cg03172931	0.04831599	-0.0272779	9.61E-08	37	19	8677774	NA	NA	NA	chr19:86743	S_Shelf	NA	NA	NA	NA	19:8677769-	Unclassified_	NA	64446
cg15684702	0.07347355	-0.0223988	9.67E-08	37	1	45297445	PTCH2;PTCH2	NM_001166	Body;Body	NA	NA	NA	NA	TRUE	NA	NA	NA	NA	289535
cg09906145	0.04461765	-0.0346942	9.67E-08	37	16	67562424	FAM65A	NM_024519	TSS1500	chr16:67562	N_Shore	NA	NA	NA	NA	16:67562352	Unclassified	TRUE	189085
cg09692396	0.04785677	-0.036153	9.69E-08	37	12	7023346	LRRC23;ENO	NM_201650;	3'UTR;TSS1500;3'UTR;	chr12:70232	Island	NA	CDMR	NA	12:6893563-	12:7023192-	Promoter_As	NA	185416
cg06061002	-0.0418961	0.02714199	9.70E-08	37	6	29638918	MOG;MOG;M	NM_206813;	3'UTR;Body;3'UTR;Bod	NA	NA	NA	NA	NA	NA	NA	NA	NA	119339
cg08824847	-0.1161813	0.14272689	9.80E-08	37	11	35052388	NA	NA	NA	NA	NA	NA	NA	TRUE	NA	NA	NA	TRUE	170243
cg04494800	0.0372296	-0.005855	9.82E-08	37	6	149775853	ZC3H12D	NM_207360	Body	chr6:149777	N_Shore	NA	NA	TRUE	NA	NA	NA	TRUE	90077
cg12882189	0.05679611	-0.0397473	9.84E-08	37	17	80402891	C17orf62;C17	NM_001100	Body;Body;Body	chr17:80401	S_Shore	NA	NA	NA	NA	NA	NA	NA	238675
cg01316378	0.07065748	-0.0473573	9.84E-08	37	10	63629580	NA	NA	NA	NA	NA	NA	RDMR	TRUE	NA	NA	NA	NA	27515
cg10205045	0.04368126	-0.0207442	9.85E-08	37	15	69087809	ANP32A	NM_006305	Body	NA	NA	NA	NA	TRUE	15:66874826	15:69087456	Promoter_As	TRUE	194409
cg05954918	-0.0588662	0.0536759	9.93E-08	37	1	21645238	ECE1	NM_001113	Body	NA	NA	NA	NA	TRUE	NA	NA	NA	NA	117401
cg21255657	0.03242378	-0.0178829	9.99E-08	37	17	77006304	CANT1;CANT	NM_138793;	TSS1500;TSS1500;TSS1	chr17:77005	S_Shore	NA	NA	NA	NA	17:77004720	NonGene_As	NA	379645
