# Supplementary material for: Impact of chemotherapy for breast cancer on leukocyte DNA methylation landscape and cognitive function: a prospective study
Source: Clin Epigenetics. 2019 Mar 12;11:45. doi: 10.1186/s13148-019-0641-1 (PMC6416954; doi:10.1186/s13148-019-0641-1)
Supplement: Supplementary file 3 — Table S3. Pathway enrichment analysis of significant CpG sites altered between pre- and post-chemotherapy with adjustment for leukocyte composition (DOCX 15 kb) [file 13148_2019_641_MOESM3_ESM.docx]

**Supplemental Table S3.** Pathway enrichment analysis of significant CpG sites altered between pre- and post-chemotherapy with adjustment for leukocyte composition

| **KEGG Term** | **P-value** | **Genes** | **Fold Enrichment** | **FDR** |
| --- | --- | --- | --- | --- |
| hsa04550: Signaling pathways regulating pluripotency of stem cells | 0.02 | *BMP2, PCGF3, JARID2, WNT3A, PIK3CD, JAK1, FZD5, TCF3, ZFHX3* | 2.7 | 0.19 |
| hsa05200: Pathways in cancer | 0.02 | *FGF18, COL4A2, BMP2, ADCY7, EPAS1, WNT3A, GNA12, PIK3CD, ITGA2, CDK6, FZD5, DAPK2, PLCG1, JAK1, PTCH2, LAMC1, RARB* | 1.8 | 0.23 |
