# Supplementary material for: Impact of chemotherapy for breast cancer on leukocyte DNA methylation landscape and cognitive function: a prospective study
Source: Clin Epigenetics. 2019 Mar 12;11:45. doi: 10.1186/s13148-019-0641-1 (PMC6416954; doi:10.1186/s13148-019-0641-1)
Supplement: Supplementary file 4 — Table S1. Descriptive characteristics of breast cancer cases and non-cancer controls. (DOCX 17 kb) [file 13148_2019_641_MOESM4_ESM.docx]

**Supplemental Table S1. Descriptive characteristics of breast cancer cases and non-cancer controls**

|  | **Cancer cases** (n=93) | **Non-cancer controls** (n=48) |
| --- | --- | --- |
|  | N (%) | N (%) |
| Age, years |  |  |
| 50-64 | 66 (71.0) | 34 (70.8) |
| 65+ | 27 (29.0) | 14 (29.2) |
| Race |  |  |
| White | 91 (97.8) | 46 (95.8) |
| Non-White | 2 (2.2) | 2 (4.2) |
| Cancer Stage |  |  |
| I | 21 (22.6) | n/a |
| II | 54 (58.1) | n/a |
| III | 16 (17.2) | n/a |
| unknown | 2 (2.2) | n/a |
| Chemotherapy setting |  |  |
| Adjuvant | 82 (88.2) | n/a |
| Neo-adjuvant | 11 (11.8) | n/a |
| Chemotherapy regimen |  |  |
| AC+T | 35 (37.6) | n/a |
| TC | 34 (36.6) | n/a |
| TP | 12 (12.9) | n/a |
| others | 12 (12.9) | n/a |
| Anthracycline |  |  |
| Yes | 47 (50.5) | n/a |
| No | 46 (49.5) | n/a |
| Total chemotherapy infusion |  |  |
| 8-11 # agents*cycles | 28 (30.1) |  |
| 12 # agents*cycles | 39 (41.9) |  |
| 14-26 # agents*cycles | 26 (28.0) |  |
| Growth factor use |  |  |
| No | 14 (15.1) | n/a |
| 1-4 cycles | 32 (34.4) | n/a |
| 5+ cycles | 47 (50.5) | n/a |
| Steroid use |  |  |
| No | 19 (20.4) | n/a |
| 1-4 cycles | 23 (24.7) | n/a |
| 5+ cycles | 51 (54.8) | n/a |

Footnote: AC+T: Adriamycin-cyclophosphamide + taxanes; TC: taxanes-cyclophosphamide; TP: taxanes + platinum
